# Human metabolism of four synthetic benzimidazole opioids: isotonitazene, metonitazene, etodesnitazene, and metodesnitazene

**DOI:** 10.1007/s00204-024-03735-0

**Published:** 2024-04-06

**Authors:** Omayema Taoussi, Diletta Berardinelli, Simona Zaami, Francesco Tavoletta, Giuseppe Basile, Robert Kronstrand, Volker Auwärter, Francesco P. Busardò, Jeremy Carlier

**Affiliations:** 1 Unit of Forensic Toxicology, Section of Legal Medicine, Department of Biomedical Sciences and Public Health, Marche Polytechnic University, Via Tronto 10/a, 60126 Ancona AN, Italy; 2 Department of Anatomical, Histological, Forensic and Orthopaedic Sciences, Sapienza University of Rome, Rome, Italy; 3 Department of Trauma Surgery, IRCCS Galeazzi Orthopedic Institute, Milan, Italy; 4 Department of Forensic Genetics and Forensic Toxicology, National Board of Forensic Medicine, Linköping, Sweden; 5 Institute of Forensic Medicine, Forensic Toxicology, Medical Center, University of Freiburg, Faculty of Medicine, University of Freiburg, Freiburg, Germany

**Keywords:** New synthetic opioids, 2-Benzylbenzimidazole opioids, Nitazenes, Metabolism, Human hepatocytes, LC-HRMS/MS

## Abstract

Following isotonitazene scheduling in 2019, the availability of alternative 2-benzylbenzimidazole opioids (nitazenes) on the global drug market increased, resulting in many fatalities worldwide. Nitazenes are potent µ-opioid receptor agonists with strong narcotic/analgesic effects, and their concentrations in biological matrices are low, making the detection of metabolite biomarkers of consumption crucial to document use in clinical and forensic settings. However, there is little to no data on the metabolism of the most recently available nitazenes, especially desnitro-analogues. The aim of the research was to assess isotonitazene, metonitazene, etodesnitazene, and metodesnitazene human metabolism and identify specific metabolite biomarkers of consumption. The four analogues were incubated with 10-donor-pooled human hepatocytes, and the incubates were analyzed by liquid chromatography-high-resolution tandem mass spectrometry and data mining with Compound Discoverer (Thermo Scientific); the analysis was supported by in silico metabolite predictions with GLORYx open-access software. Metabolites were identified in postmortem blood and/or urine samples from two metonitazene-positive and three etodesnitazene-positive cases following the same workflow, with and without glucuronide hydrolysis in urine, to confirm in vitro results. Twelve, nine, twenty-two, and ten metabolites were identified for isotonitazene, metonitazene, etodesnitazene, and metodesnitazene, respectively. The main transformations were *N*-deethylation at the *N*,*N*-diethylethanamine side chain, *O*-dealkylation, and further *O*-glucuronidation. In vitro and autopsy results were consistent, demonstrating the efficacy of the 10-donor-pooled human hepatocyte model to predict human metabolism. We suggest the parent and the corresponding *O*-dealkyl- and *N*-deethyl-*O*-dealkyl metabolites as biomarkers of exposure in urine after glucuronide hydrolysis, and the corresponding *N*-deethyl metabolite as additional biomarker in blood.

**Supplementary Information:**

The online version contains supplementary material available at 10.1007/s00204-024-03735-0.

## Introduction

In the early 2010s, the USA was struck by the “opioid crisis”, as illicit fentanyl and new synthetic opioids (NSOs) flooded the illegal drug market (Prekupec et al. 2017). As a result, the number of opioid-related overdose deaths sharply increased in the USA from 2013 onwards, mainly involving fentanyl and analogues (Mattson et al. 2021), and the fatal trend spread worldwide shortly after (UNODC 2020; Brunetti et al. 2021). Subsequent to the scheduling of fentanyl analogues in the USA and China in 2019, users turned to other NSO subclasses including benzimidazole opioids (nitazene analogues) (Bao et al. 2019; Papsun et al. 2022; DEA 2023; Barrios et al. 2024).

Benzimidazole opioids are *µ*-opioid receptor (MOR) agonists with antinociceptive properties. Although the potency of some of these compounds is similar to that of morphine, the pharmacological activity of several analogues, such as etonitazene, isotonitazene, metonitazene, and etonitazepyne, is similar or even much higher compared to that of the medically used, highly potent opioid fentanyl (Hunger et al. 1957, 1960; Vandeputte et al. 2021, 2022a). As for traditional opioids, miosis, fatigue, reduced consciousness, nausea, vomiting, cyanosis, and respiratory depression are common adverse effects (Ujváry et al. 2021), and hundreds of intoxications involving benzimidazole opioids were reported (Ujváry et al. 2021; Montanari et al. 2022; Papsun et al. 2022; DEA 2023). Dozens of isotonitazene-related fatalities were reported in 2019, until its scheduling in June 2020 in the USA, and dozens of metonitazene-related deaths were notified from November 2020 onwards (DEA 2018; Montanari et al. 2022). With the decrease of isotonitazene popularity, several new analogues emerged onto the drug market, and ten are currently tracked through the European Early Warning System (EMCDDA 2022). According to the European Drug Report 2023, the European Monitoring Centre for Drugs and Drug Addiction (EMCDDA) has issued ten official notifications connected to benzimidazole opioid use since 2019 (EMCDDA 2023a). New analogues, i.e., butonitazene, etodesnitazene, etonitazepyne, etonitazepipne, flunitazene, metodesnitazene, and protonitazene, emerged in 2021 and 2022 (Vandeputte et al. 2022b, c; Schumann et al. 2023; CFSRE 2024); fatalities and/or intoxications involving butonitazene, flunitazene, etonitazepyne, and etonitazepipne were also reported (Vandeputte et al. 2022b; Calello et al. 2022; Montanari et al. 2022; Di Trana et al. 2023). In biological matrices, benzimidazole opioid concentrations are low, typically below 10 ng/mL in blood, and they are almost always detected along with other central nervous system depressants such as benzodiazepines (“benzo dope” recent deadly trend mixing opioids and benzodiazepines) and other opioids (Montanari et al. 2022; EMCDDA 2023b). Considering their lack of specific symptoms and challenging detection in analytical toxicology, benzimidazole opioid-related intoxications and deaths are probably underestimated from an epidemiological point of view.

Metabolite detection is critical to document drug consumption in clinical and forensic settings, and phase I metabolites of isotonitazene and metonitazene have been identified in authentic cases of consumption (Krotulski et al. 2020, 2021). However, the focus was on reporting isotonitazene and metonitazene concentrations, and phase II metabolites were not investigated. Metabolite detection is also crucial since they can be active. For example, isotonitazene major metabolites 4′-hydroxyl-nitazene, 5-amino-isotonitazene, and *N*-deethyl-isotonitazene are biologically active, and *N*-deethyl-isotonitazene proved more even potent than the parent in in vitro experiments evaluating MOR activation (Vandeputte et al. 2021). Etodesnitazene major phase I metabolites were recently identified in rats (Grigoryev et al. 2023). The metabolic fate of other benzimidazole opioids was not yet assessed.

To continue these efforts to describe the metabolism of emerging opioids, the aim of the present study was to investigate the human metabolism of isotonitazene and metonitazene in human hepatocyte incubations as well as in positive samples from postmortem cases to confirm the previously published data, include phase II metabolites, and verify suitability of the in vitro model for predicting the metabolism of benzimidazole opioids in humans. This model was then applied to etodesnitazene and metodesnitazene to identify suitable metabolite biomarkers of consumption.

## Materials and methods

### Chemicals and reagents

Isotonitazene, metonitazene, etodesnitazene, metodesnitazene, 4′-hydroxyl-nitazene, *N*-deethyl-isotonitazene, and 5-amino-isotonitazene were purchased from Cayman Chemical (Ann Harbor, MI, USA) and solubilized in methanol. Diclofenac was purchased from Sigma Aldrich (Milan, Italy) and solubilized in methanol. Standard solutions were stored at − 20 °C prior to analysis.

LC–MS grade water, acetonitrile, and methanol were obtained from Carlo Erba (Cornaredo, Italy), and LC–MS grade formic acid was obtained from Sigma Aldrich. Reagent grade ammonium acetate from Levanchimica (Bari, Italy) and LC grade acetic acid from Sigma Aldrich were used to prepare 10 mol/L ammonium acetate, pH 4.5. β-Glucuronidase (100,000–200,000 units/mL) from limpets (*Patella vulgata* L.) was purchased from Sigma Aldrich.

Thawing medium and cryopreserved 10-donor-pooled human hepatocytes were purchased from Lonza (Basel, Switzerland). l-Glutamine, HEPES (2-[4-(2-hydroxyethyl)-1-piperazinyl]ethanesulfonic acid), and Williams’ medium E were bought from Sigma Aldrich. l-Glutamine and HEPES were dissolved in Williams’ medium E to 2 and 20 mmol/L, respectively; the supplemented medium (sWME) was prepared extemporaneously.

### In silico metabolite prediction

Phase I and phase II putative metabolites of isotonitazene, metonitazene, etodesnitazene, and metodesnitazene were predicted with GLORYx freeware (De Bruyn Kops et al. 2021). First-generation metabolites with a prediction score equal or higher than 50% were reprocessed to simulate second-generation metabolism. The score of second-generation metabolites was multiplied to the score of the corresponding first-generation metabolite to calculate an “adjusted score”.

### Human hepatocyte incubations

Incubations were conducted as previously described (Di Trana et al. 2021). Briefly, the hepatocytes were thawed in thawing medium at 37 °C, which was then discarded to resuspend the cells in sWME. Each analogue was diluted to 20 µmol/L in sWME and 250 µL was incubated with 250 µL 2 × 10^6^ viable hepatocytes/mL for 0 or 3 h at 37 °C; each analogue was incubated separately. Negative controls, i.e., hepatocytes in sWME without the drugs and each drug separately in sWME without hepatocytes, were incubated for 0 and 3 h in the same conditions. Diclofenac also was incubated in the same conditions to ensure proper metabolic activity throughout the experiments. Reactions were quenched with ice-cold acetonitrile and centrifugation. The incubates were stored at − 80 °C until analysis.

### Postmortem samples

Postmortem biological samples from five nitazene-positive cases were analyzed. Case #1 (Sweden) was positive for metonitazene; blood and urine were collected. Case #2 (Munich, Germany) was positive for metonitazene; blood was collected; metonitazene was not quantified, but JWH-210, THJ-2201, 4-fluoromethylphenidate, oxycodone, noroxycodone, and naloxone were also detected; acute polydrug intoxication was determined as the cause of death. Cases #3 and #4 (Sweden) were positive for etodesnitazene; urine was collected in both cases. Case #5 (Munich, Germany) was positive for etodesnitazene; blood was collected; etodesnitazene concentration was 3.5 ng/mL, and 2-aminoindane and *N*-methyl-2-aminoindane were also detected; acute polydrug intoxication was determined as the cause of death.

### Sample preparation

#### Incubates

After thawing at room temperature, 100 µL sample was mixed with 100 µL acetonitrile and centrifuged for 10 min, 15,000*g*, at room temperature. The supernatants were dried under nitrogen and reconstituted in 150 µL 0.1% formic acid in water:0.1% formic acid in acetonitrile 90:10 (v/v). After centrifugation for 10 min, 15,000*g*, at room temperature, the supernatants were transferred into autosampler vials with a glass insert.

#### Urine and blood samples

Samples were thawed at room temperature, and 100 µL was mixed with 200 µL acetonitrile and centrifuged for 10 min, 15,000*g*, at room temperature. The supernatants were evaporated to dryness under nitrogen at 37 °C. The residues were reconstituted in 150 µL 0.1% formic acid in water:0.1% formic acid in acetonitrile 90:10 (v/v). After centrifugation for 10 min, 15,000*g*, at room temperature, the supernatants were transferred into autosampler vials with a glass insert.

At the same time, to study metabolite glucuronidation, 100 µL urine was mixed with 10 µL 10 mol/L ammonium acetate, pH 5.0, and 100 µL β-glucuronidase (5000 units) in conical glass tubes. Tubes were capped and incubated at 37 °C. After 90 min, 400 µL ice-cold acetonitrile was added to the mixtures, which were then mixed and centrifuged for 10 min, 15,000*g*, at room temperature. The supernatants were evaporated to dryness under nitrogen at 37 °C. The residues were reconstituted in 150 µL 0.1% formic acid in water:0.1% formic acid in acetonitrile 90:10 (v/v). After centrifugation for 10 min, 15,000*g*, at room temperature, the supernatants were transferred into autosampler vials with a glass insert. The samples were also prepared in the same conditions with 100 µL water instead of β-glucuronidase to ensure that the hydrolysis was enzymatic.

### Liquid chromatography-high-resolution tandem mass spectrometry settings

Extracted samples were kept in an autosampler at 10 ± 1 °C, before injecting 15 µL onto the chromatographic system. LC-HRMS/MS analysis was performed with a Dionex UltiMate 3000 chromatographic system coupled with a Thermo Scientific (Waltham, MA, USA) Q Exactive mass spectrometer equipped with a heated electrospray ionization (HESI) source. Each sample was injected once in positive- and once in negative-ionization mode. LC-HRMS/MS settings were the same for each analogue to facilitate comparison.

#### Liquid chromatography

Separation was performed through a Kinetex Biphenyl column (150 × 2.1 mm, 2.6 µm) from Phenomenex (Castel Maggiore, Italy) with a mobile phase gradient composed of 0.1% formic acid in water (mobile phase A, MPA), and 0.1% formic acid in acetonitrile (mobile phase B, MPB) at 37 °C. The total run time was 30 min for a 0.4 mL/min flow rate. The gradient started with 5% MPB for 2 min, and increased to 25% within 12 min, to 50% within 5 min, then to 95% within 1 min; 95% MPB was maintained for 5 min, before returning to initial conditions within 0.1 min; re-equilibration time was 4.9 min.

#### High-resolution tandem mass spectrometry

HESI source settings were optimized using isotonitazene, metonitazene, etodesnitazene, metodesnitazene, 4′-hydroxyl-nitazene, and 5-amino-isotonitazene signal after injecting the standards at 1 µg/mL in MPA:MPB 90:10 (v/v): spray voltage, ± 3.5 kV; sheath gas flow rate, 50 a.u.; auxiliary gas flow rate, 10 a.u.; auxiliary gas temperature, 300 °C; capillary temperature, 300 °C; S-lens radio frequency level, 50 a.u.

Data were acquired in full-scan HRMS/data-dependent HRMS/MS modes (FullMS/ddMS^2^). FullMS settings were: resolution, 70,000 (full width at half maximum at *m/z* 200), mass range, *m/z* 250–720, automatic gain control (AGC) target, 10^6^; max injection time (IT), 0.2 s. ddMS^2^ settings were: resolution, 17,500; topN, 5 (pick others if idle); intensity threshold, 10^4^; dynamic exclusion, 2 s; isolation window, *m/z* 1.2; normalized collision energy, 40, 70, and 80%; AGC target, 2 × 10^5^; max IT, 0.064 s. A different inclusion list was used for each analogue, based on in silico predictions (Supplementary Table S1) and postulation (Krotulski et al. 2020, 2021) (Table S2), but the same exclusion list was used, based on background ions. The orbitrap was calibrated prior to analysis (*m/z* 74.0964–1321.9843 and *m/z* 68.9958–1379.9908 in positive- and negative-ionization modes, respectively) and a lock mass list with previously identified background ions was used throughout the analysis for better accuracy (Keller et al. 2008).

### Metabolite identification

LC-HRMS/MS data were processed with Thermo Scientific Compound Discoverer in a single analysis, as previously described (Di Trana et al. 2021). Briefly, the ions were compared to a list of theoretical metabolites generated according to the settings displayed in Table S3 (intensity threshold, 5 × 10^3^; HRMS mass tolerance, 5 ppm). Besides, the HRMS/MS spectra and theoretical elemental composition of the ions were compared to mzCloud (Drugs of Abuse/Illegal Drugs database), ChemSpider (Cayman Chemical, DrugBank), and HighResNPS online databases (intensity threshold, 10^5^; HRMS mass tolerance, 5 ppm; HRMS/MS mass tolerance; 10 ppm).

### 4′-Hydroxyl-nitazene, *N*-deethyl-isotonitazene, and 5-amino-isotonitazene identification

4′-Hydroxyl-nitazene, *N*-deethyl-isotonitazene, and 5-amino-isotonitazene standards were diluted to 1 µg/mL in MPA:MPB (90:10, v/v) and analyzed in the same LC-HRMS/MS conditions to confirm their detection in incubates and samples.

## Results

### In silico metabolite prediction

Human metabolite predictions are presented in Supplementary Table S1. A total of ten first-generation (pA1 to pA10 by ascending prediction score) and 45 s-generation (pAX-1 to pAX-14 by ascending prediction score, pAX representing the corresponding first-generation metabolite) metabolites were predicted for isotonitazene; 13 metabolites were duplicates. A total of 11 first-generation (pB1 to pB11) and 40 s-generation (pBX-1 to pBX-10) metabolites were predicted for metonitazene; 10 metabolites were duplicates. A total of 10 first-generation (pC1 to pC10) and 40 s-generation (pCX-1 to pCX-12) metabolites were predicted for etodesnitazene; 15 metabolites were duplicates. A total of 8 first-generation (pD1 to pD8) and 35 s-generation (pDX-1 to pDX-10) metabolites were predicted for metodesnitazene; 10 metabolites were duplicates. First-generation metabolites for all four nitazenes were produced by *N*-deethylation, hydroxylation, *N*-oxidation, *O*-dealkylation, and oxidative deamination; second-generation transformations further included *O*- glucuronidation and *O*-sulfation. Isotonitazene and metonitazene had 10 *O*-dealkyl metabolites in common, and etodesnitazene and metodesnitazene shared 9 *O*-dealkyl metabolites.

To assist in LC-HRMS/MS analysis, all the predicted metabolites were included in the LC-HRMS/MS inclusion list (Supplementary Table S2). All predicted metabolic reactions and combinations were included in the list of possible transformations for data mining.

### HRMS/MS fragmentation pattern of isotonitazene, metonitazene, etodesnitazene, and metodesnitazene

All four compounds were detected only in positive-ionization mode under the applied analytical conditions.

**Fig. 1:**
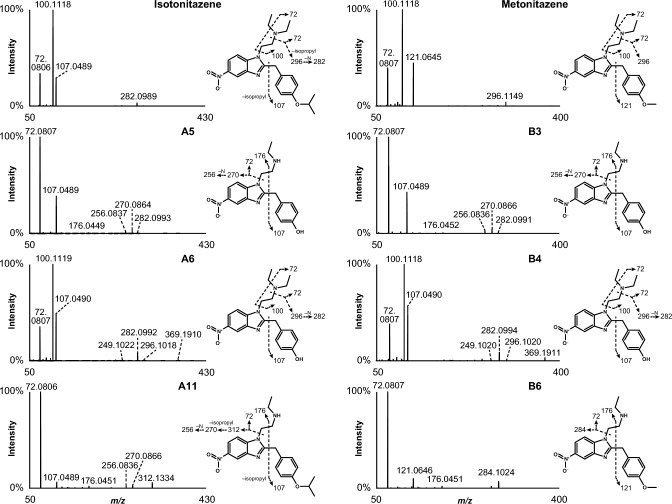
HRMS/MS spectra of isotonitazene, metonitazene, and major metabolites identified in human hepatocyte incubates and authentic postmortem samples as well as suggested fragmentation patterns. A5 and B3 are the same metabolite; A6 and B4 are the same metabolite

**Fig. 2:**
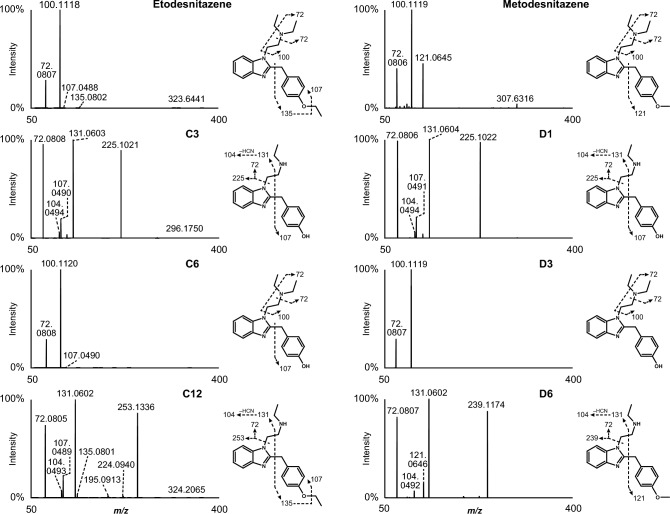
HRMS/MS spectra of etodesnitazene, metodesnitazene, and major metabolites identified in human hepatocyte incubates and authentic postmortem samples as well as suggested fragmentation patterns. C3 and D1 are the same metabolite; C6 and D3 are the same metabolite

As expected from structural analogues, they displayed similar fragmentation patterns (Figs. 1 and 2). For the four analogues, the most intense fragments corresponded to the *N*,*N*-diethylethanamine (*m/z* 100.1121 ± 5 ppm) and diethylamine (*m/z* 72.0808 ± 5 ppm) groups. Fragments at *m/z* 121.0645 in metonitazene and metodesnitazene and *m/z* 135.0802 in etodesnitazene corresponded to the 1-methyl-4’-alkoxybenzyl sidechain, which was further fragmented to *m/z* 107.0491 ± 5 ppm in isotonitazene and etodesnitazene due to isopropyl and ethyl loss, respectively. In metonitazene fragmentation pattern, an additional minor fragment was produced through α-cleavage at the nitrogen atom of the diethylamine side chain and nitrogen loss from the nitro group (*m/z* 296.1149), which was further fragmented in isotonitazene due to isopropyl loss (*m/z* 282.0989).

### Metabolite identification in human hepatocyte incubations

**Fig. 3:**
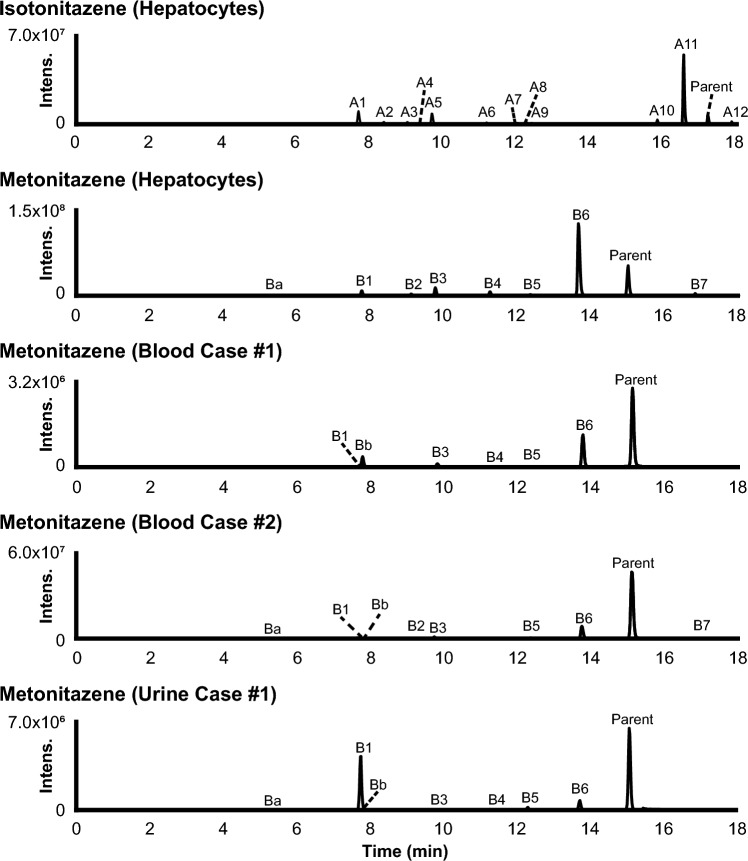
Extracted-ion chromatograms of isotonitazene, metonitazene, and metabolites identified in human hepatocyte incubates and authentic postmortem samples. Mass tolerance, 5 ppm

**Fig. 4:**
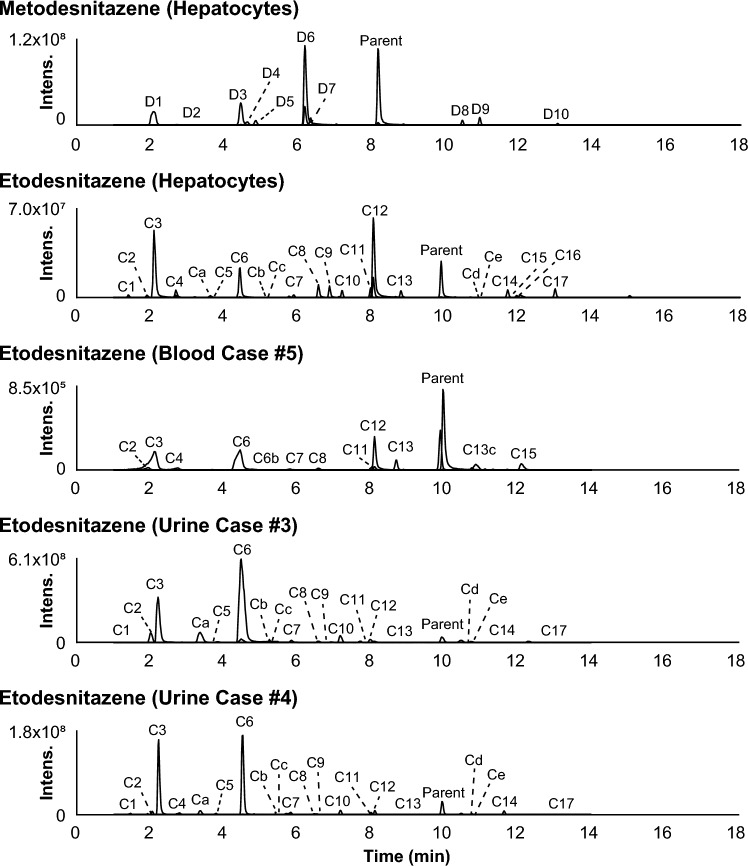
Extracted-ion chromatograms of etodesnitazene, metodesnitazene, and metabolites identified in human hepatocyte incubates and authentic postmortem samples. Mass tolerance, 5 ppm

LC-HRMS data were automatically mined to produce a list of potential metabolites that were manually checked by the operators. Isotonitazene LC-HRMS area was 2.5 × 10^9^ and 5.5 × 10^7^ in the 0 and 3 h incubates with hepatocytes, respectively; 12 metabolites were identified (A1 to A12 by ascending retention time) after 3 h of incubation with human hepatocytes (Fig. 3). Metonitazene LC-HRMS area was 1.4 × 10^9^ and 2.1 × 10^8^ in the 0 and 3 h incubates with hepatocytes, respectively; 7 metabolites were identified (B1 to B7) after 3 h of incubation with human hepatocytes (Fig. 3). Etodesnitazene LC-HRMS area was 1.0 × 10^9^ and 1.2 × 10^8^ in the 0 and 3 h incubates with hepatocytes, respectively; 17 metabolites were identified (C1 to C17) after 3 h of incubation with human hepatocytes (Fig. 4). Metodesnitazene LC-HRMS area was 1.6 × 10^9^ and 5.3 × 10^8^ in the 0 and 3 h incubates with hepatocytes, respectively; 10 metabolites were identified (D1 to D10) after 3 h of incubation with human hepatocytes (Fig. 4). The elemental composition, retention time, accurate mass of molecular ion and deviation from theoretical mass, and LC-HRMS peak area of parents and metabolites in positive-ionization mode after 3 h incubation with hepatocytes are reported in Tables 1, 2. For all four analogues, major metabolic transformations were *N*-deethylation, *O*-dealkylation, and further *O*-glucuronidation. Minor reactions included hydroxylation, oxidative deamination to alcohol or carboxylic acid, dehydrogenation, and ketone formation. 5-Amino-isotonitazene, produced through isotonitazene nitro reduction, the structure of which was confirmed through comparison with the commercially available reference standard, was also found, but with an intensity below the detection threshold. Metabolites’ signal was always higher in positive-ionization mode, and negative ionization did not bring more information for structure elucidation. All data are therefore reported in the positive-ionization mode in this article. HRMS/MS spectra of major metabolites are displayed in Figs. 1 and 2. The structure elucidation of major metabolites is described below.

**Table 1:** Metabolic transformation, elemental composition, retention time (RT), accurate mass of molecular ion, deviation from theoretical accurate mass, and liquid chromatography-high-resolution mass spectrometry peak area of isotonitazene and metabolites in positive-ionization mode after 3-h Incubation with human hepatocytes

ID	Biotransformation	Elemental composition	RT, min	*m/z* (Δppm)	Peak area in 3-h incubate
A1	*N*-Deethylation + *O*-Deisopropylation + *O*-Glucuronidation	C_24_H_28_N_4_O_9_	7.69	517.1923 (− 1.13)	1.1 × 10^8^
A2	*N*-Deethylation + *N*-Deethylation + *O*-Deisopropylation	C_16_H_16_N_4_O_3_	8.39	313.1292 (− 0.88)	1.7 × 10^7^
A3	*O*-Deisopropylation + *O*-Glucuronidation	C_26_H_32_N_4_O_9_	9.03	545.2243 (0.17)	1.7 × 10^7^
A4	*O*-Deisopropylation + Hydroxylation (phenylmethyl) + *O*-Glucuronidation	C_26_H_32_N_4_O_10_	9.37	561.2188 (− 0.66)	1.5 × 10^7^
A5	*N*-Deethylation + *O*-Deisopropylation	C_18_H_20_N_4_O_3_	9.69	341.1603 (− 1.40)	8.8 × 10^7^
A6	*O*-Deisopropylation	C_20_H_24_N_4_O_3_	11.19	369.1920 (− 0.34)	1.3 × 10^7^
A7	*N*-Deethylation + Hydroxylation (*O*-isopropyl)	C_21_H_26_N_4_O_4_	12.02	399.2023 (− 0.96)	1.1 × 10^7^
A8	*O*-Deisopropylation + Oxidative deamination	C_16_H_15_N_3_O_4_	12.27	314.1134 (− 0.52)	1.7 × 10^7^
A9	*N*-Deethylation + *O*-Deisopropylation + O –2H (*N*-alkyl)	C_18_H_18_N_4_O_4_	12.41	355.1400 (− 0.29)	1.2 × 10^7^
A10	*N*-Deethylation + *N*-Deethylation	C_19_H_22_N_4_O_3_	15.84	355.1760 (− 1.23)	3.3 × 10^7^
A11	*N*-Deethylation	C_21_H_26_N_4_O_3_	16.56	383.2072 (− 1.45)	4.4 × 10^8^
Isotonitazene (parent)		C_23_H_30_N_4_O_3_	17.21	411.2386 (− 1.09)	5.5 × 10^7^
A12	Oxidative dealkylation + ω-Carboxylation + *O*-Glucuronidation	C_28_H_34_N_4_O_11_	17.86	603.2296 (− 0.22)	1.7 × 10^7^

Mass tolerance, 5 ppm

**Table 2:** Metabolic transformation, elemental composition, retention time (RT), accurate mass of molecular ion, deviation from theoretical accurate mass, and liquid chromatography-high-resolution mass spectrometry peak area of metonitazene and metabolites in positive-ionization mode after 3-h Incubation with human hepatocytes and in postmortem samples

ID	Biotransformation	Elemental composition	RT, min	*m/z* (Δppm)	Peak area in 3-h incubates	Blood Case #1	Blood Case #2	Peak area in urine Case #1	
								Without hydrolysis	With hydrolysis
Ba	Nitro reduction	C_21_H_29_N_4_O	5.52	353.2336 (0.03)	9.7 × 10^5*^	ND	2.4 × 10^6^	2.6 × 10^6^	2.1 × 10^6^
B1	*N*-Deethylation + *O*-Demethylation + *O*-Glucuronidation	C_24_H_28_N_4_O_9_	7.76	517.1926 (− 0.58)	3.0 × 10^7^	1.5 × 10^6^	1.8 × 10^6^	1.7 × 10^7^	ND
Bb	Nitro reduction + *N*-Acetylation	C_23_H_30_N_4_O_2_	7.7	395.2442 (0.12)	9.2 × 10^5*^	1.8 × 10^6^	9.4 × 10^5^	1.0 × 10^5^	8.0 × 10^4^
B2	*O*-Demethylation + *O*-Glucuronidation	C_26_H_32_N_4_O_9_	9.11	545.2241 (− 0.16)	1.0 × 10^7^	ND	ND	ND	ND
B3	*N*-Deethylation + *O*-Demethylation	C_18_H_20_N_4_O_3_	9.77	341.1606 (− 0.63)	5.2 × 10^7^	5.4 × 10^5^	4.5 × 10^6^	ND	2.7 × 10^7^
B4	*O*-Demethylation	C_20_H_24_N_4_O_3_	11.27	369.1919 (− 0.58)	2.6 × 10^7^	1.6 × 10^6^	ND	1.4 × 10^5^	2.8 × 10^6^
B5	*O*-Demethylation + Oxidative deamination	C_16_H_15_N_3_O_4_	12.36	314.1134 (− 0.42)	8.1 × 10^6^	1.5 × 10^4^	1.8 × 10^6^	8.9 × 10^5^	4.2 × 10^6^
B6	*N*-Deethylation	C_19_H_22_N_4_O_3_	13.66	355.1759 (− 1.59)	5.5 × 10^8^	5.6 × 10^6^	4.1 × 10^7^	3.3 × 10^6^	5.8 × 10^6^
Metonitazene (parent)		C_21_H_26_N_4_O_3_	15.02	383.2073 (− 1.29)	2.1 × 10^8^	1.5 × 10^7^	2.5 × 10^8^	2.8 × 10^7^	2.8 × 10^7^
B7	*O*-Demethylation + Oxidation (Alkyl) + Desaturation (Alkyl)	C_19_H_20_N_4_O_4_	16.84	369.1561 (0.99)	1.1 × 10^7^	ND	5.3 × 10^5^	ND	ND

Mass tolerance, 5 ppm; *ND* not detected

^*^Found retrospectively in incubates after identification in authentic samples

#### *N*-deethylation

*N*-deethylation at the *N*,*N*-diethylethanamine side chain was preponderant in isotonitazene (A11), metonitazene (B6), etodesnitazene (C12), and metodesnitazene (D6) in vitro metabolism. The ethyl loss (–C_2_H_4_) was substantiated by a − 28.0313 Da ± 5 ppm mass shift from the parent drug. The lack of major fragment at *m/z* 100.1121 ± 5 ppm (*N*,*N*-diethylethanamine group) in A11, B6, C12, and D6 HRMS/MS spectra further indicated that the reaction occurred at the *N*,*N*-diethylethanamine side chain, which was supported by fragments at *m/z* 312.1334 (A11), 284.1024 (B6), 253.1336 (C12), and 239.1174 (D6), produced by diethylamine chain loss, confirming that the benzimidazole core and the 1-methyl-4′-alkoxybenzyl sidechain were not modified during metabolization. A11 structure (*N*-deethyl-isotonitazene) was confirmed through the injection of the reference standard, which was commercially available at the time of the study.

Further minor reactions included another *N*-deethylation to the corresponding *N*,*N*-dideethyl metabolites and subsequent oxidative deamination.

#### *O*-dealkylation and further *O*-glucuronidation

*O*-dealkylation was also a major transformation in metonitazene (B4), etodesnitazene (C6), and metodesnitazene (D3) metabolism, but was relatively less intense in isotonitazene (A6). Isotonitazene and metonitazene structural difference being carried by the alkoxy side chain, A6 and B4 were the same molecules; metabolites C6 from etodesnitazene and D3 from metodesnitazene were also the same molecules. Propyl (–C_3_H_6_), methyl (–CH_2_), and ethyl (–C_2_H_4_) losses were demonstrated by a − 42.0466 Da mass shift from isotonitazene, − 14.0157 Da ± 5 ppm mass shift from metonitazene and metodesnitazene, and − 28.0308 Da mass shift from etodesnitazene, respectively. B4 and D3 HRMS/MS spectra displayed a major fragment at *m/z* 107.0491 ± 5 ppm instead of *m/z* 121.0645 detected in parents’ spectra, indicating *O*-demethylation at the methoxy group. A6 and C6 fragmentation patterns were similar to those of isotonitazene and etodesnitazene (without fragment at *m/z* 135.0802), respectively, pointing towards *O*-dealkylation at the alkoxy group. Remarkably, *O*-dealkylation greatly decreased retention on the biphenyl column for all four analogues. A6/B4 structure (4′-hydroxyl-nitazene) was confirmed through the injection of the commercially available reference standard.

Subsequent *O*-glucuronidation of A6/B4 and C6/D3 to A3/B2 and C4/D2, respectively, was detected, albeit with minor signal intensity. A6/B4 and C6/D3 fragmentation patterns were similar to those of A3/B2 and C4/D2, respectively.

#### Combination of *N*-deethylation and *O*-dealkylation and further *O*-glucuronidation

Combination of *N*-deethylation and *O*-dealkylation produced major metabolites of isotonitazene (A5), metonitazene (B3), etodesnitazene (C3), and metodesnitazene (D1), with an intensity similar or higher than that of the corresponding *O*-dealkyl metabolites. As *O*-dealkyl metabolites, A5 and B3 were the same molecules, as were C3 and D1. Ethyl loss (–C_2_H_4_) plus propyl (–C_3_H_6_), methyl (–CH_2_), or ethyl (–C_2_H_4_) losses were demonstrated by a − 70.0783 Da mass shift from isotonitazene, − 42.0470 Da ± 5 ppm mass shift from metonitazene and metodesnitazene, and − 56.0621 Da mass shift from etodesnitazene, respectively. The position of the deethylation at the *N*,*N*-diethylethanamine side chain and the dealkylation at the alkoxy group was confirmed through A5/B3 and C3/D1 fragmentation, as previously detailed.

A5/B3 and C3/D1 underwent further *O*-glucuronidation to A1/B1 and C1; *N*-deethyl-*O*-demethyl-metodesnitazene glucuronide was detected with a signal intensity below the established threshold. A1/B1 and C1 *N*-deethylation (–C_2_H_4_), *O*-dealkylation [deisopropylation (–C_3_H_6_), demethylation (–CH_2_), or deethylation (–C_2_H_4_)], and *O*-glucuronidation (+ C_6_H_8_O_6_) was shown by the mass shift from the corresponding parent (+ 105.9537 Da, + 133.9853 Da, and + 119.9701 Da for A1, B1, and C1, respectively) and similarity of fragmentation with A5/B3 and C3/D1, respectively. Remarkably, C1 eluted at the very beginning of the LC gradient, demonstrating the importance of LC setting optimization for metabolite identification studies to avoid missing more polar metabolites.

### Metabolite identification in postmortem samples

Blood (case #1) and blood and urine (case #2) samples from metonitazene-positive cases were analyzed (Table 3). Out of 7 metabolites identified in vitro, 6 were detected in either blood or urine: all metabolites identified in vitro were found in both blood samples beside B2 and B7 in case #1, and B2 and B4 in case #2; B2, B3, and B7 were not detected in the non-hydrolyzed urine, and B1 (glucuronide), B2 (glucuronide), and B7 were not detected in hydrolyzed urine. Two additional metabolites, produced through nitro reduction (− 2O + 2H) (Ba) and further *N*-acetylation (+ C_2_H_2_O) (Bb) were detected in all samples, although they were not major metabolites. Ba and Bb were retrospectively detected in incubates, but with a signal intensity below the established threshold. *N*-Deethyl-metonitazene (B6) and metonitazene itself were dominant in both blood samples; and *O*-demethyl-metonitazene (B4), *N*-deethyl-*O*-demethyl-metonitazene (B3), and parent were major in hydrolyzed urine.

**Table 3:** Metabolic transformation, elemental composition, retention time (RT), accurate mass of molecular ion, deviation from theoretical accurate mass, and liquid chromatography-high-resolution mass spectrometry peak area of etodesnitazene and metabolites in positive-ionization mode after 3-h Incubation with human hepatocytes and in postmortem samples

ID	Biotransformation	Elemental composition	RT, min	*m/z* (Δppm)	Peak area in 3-h incubates	Blood case #5	Peak area in urine			
							Urine case #3		Urine case #4	
							Without hydrolysis	With hydrolysis	Without hydrolysis	With hydrolysis
C1	*N*-Deethylation + *O*-Deethylation + *O*-Glucuronidation	C_24_H_29_N_3_O_7_	1.39	472.2080 (0.33)	1.9 × 10^7^	ND	7.4 × 10^8^	1.3 × 10^6^	1.5 × 10^8^	1.5 × 10^7^
C2	*N*-Deethylation + Hydroxylation	C_20_H_25_N_3_O_2_	1.90	340.2019 (− 0.07)	7.0 × 10^6^	2.3 × 10^5^	1.5 × 10^8^	3.9 × 10^8^	1.3 × 10^7^	2.3 × 10^7^
C3	*N*-Deethylation + *O*-Deethylation	C_18_H_21_N_3_O	2.10	296.1758 (0.24)	2.5 × 10^8^	2.9 × 10^6^	7.3 × 10^8^	2.1 × 10^9^	2.8 × 10^8^	6.5 × 10^8^
C4	*O*-Deethylation + *O*-Glucuronidation	C_26_H_33_N_3_O_7_	2.69	500.2395 (0.81)	2.5 × 10^7^	2.5 × 10^5^	4.5 × 10^9^	7.4 × 10^6^	2.8 × 10^8^	2.6 × 10^7^
Ca	*N*-Deethylation + Hydroxylation	C_20_H_25_N_3_O_2_	3.21	340.2019 (− 0.05)	2.2 × 10^6*^	ND	1.1 × 10^7^	6.3 × 10^8^	2.0 × 10^6^	5.1 × 10^7^
C5	*N*-Deethylation + Hydroxylation (benzimidazole)	C_20_H_25_N_3_O_2_	3.63	340.2020 (0.05)	6.9 × 10^6^	ND	1.7 × 10^7^	2.5 × 10^7^	9.1 × 10^6^	1.3 × 10^7^
C6	*O*-Deethylation	C_20_H_25_N_3_O	4.43	324.2071 (0.25)	1.0 × 10^8^	2.4 × 10^6^	1.3 × 10^9^	6.0 × 10^9^	1.1 × 10^8^	7.9 × 10^8^
Cb	*O*-Deethylation + Oxidative deamination + *O*-Glucuronidation	C_22_H_24_N_2_O_8_	5.39	445.1605 (− 0.09)	2.5 × 10^6*^	ND	7.1 × 10^7^	ND	2.8 × 10^7^	2.7 × 10^6^
Cc	Hydroxylation (*O*-ethyl)	C_22_H_29_N_3_O_2_	5.76	368.2331 (− 0.14)	3.9 × 10^6*^	6.1 × 10^4^	2.4 × 10^7^	6.6 × 10^7^	4.0 × 10^6^	8.1 × 10^6^
C7	*O*-Deethylation + Oxidative deamination + ω-Carboxylation	C_16_H_14_N_2_O_3_	5.89	283.1075 (− 0.84)	7.5 × 10^6^	5.5 × 10^5^	6.7 × 10^7^	5.6 × 10^7^	3.8 × 10^7^	3.8 × 10^7^
C8	*N*-Deethylation + Hydroxylation (benzimidazole)	C_20_H_25_N_3_O_2_	6.56	340.2016 (− 0.95)	4.1 × 10^7^	1.1 × 10^5^	1.2 × 10^7^	4.4 × 10^7^	7.1 × 10^6^	1.6 × 10^7^
C9	*N*-Deethylation + *N*-Deethylation	C_18_H_21_N_3_O	6.86	296.1756 (− 0.47)	3.5 × 10^7^	ND	1.1 × 10^6^	2.1 × 10^6^	7.7 × 10^5^	1.1 × 10^6^
C10	*O*-Deethylation + Oxidative deamination	C_16_H_16_N_2_O_2_	7.19	269.1283 (− 0.46)	1.9 × 10^7^	ND	4.2 × 10^7^	2.6 × 10^8^	2.9 × 10^6^	3.9 × 10^7^
C11	Hydroxylation (benzimidazole or *O*-ethyl)	C_22_H_29_N_3_O_2_	7.97	368.2330 (− 0.61)	2.6 × 10^7^	1.1 × 10^5^	1.6 × 10^7^	1.1 × 10^8^	3.8 × 10^6^	1.7 × 10^7^
C12	*N*-Deethylation	C_20_H_25_N_3_O	8.05	324.2066 (− 1.35)	3.1 × 10^8^	1.7 × 10^6^	2.1 × 10^7^	4.7 × 10^7^	1.6 × 10^7^	3.4 × 10^7^
C13	*N*-Deethylation + Terminal desaturation (*O*-ethyl)	C_20_H_23_N_3_O	8.80	322.1911 (0.90)	2.2 × 10^7^	4.8 × 10^5^	1.6 × 10^6^	3.5 × 10^6^	4.7 × 10^5^	1.3 × 10^6^
Etodesnitazene (parent)		C_22_H_29_N_3_O	9.89	352.2379 (− 1.39)	1.2 × 10^8^	4.5 × 10^6^	2.5 × 10^8^	2.3 × 10^8^	1.5 × 10^8^	1.3 × 10^8^
Cd	*O*-Deethylation + O –2H (*N*-alkyl)	C_20_H_23_N_3_O_2_	10.46	338.1863 (0.00)	1.6 × 10^6*^	ND	3.8 × 10^7^	1.0 × 10^8^	2.1 × 10^6^	1.6 × 10^7^
Ce	Hydroxylation (benzimidazole)	C_22_H_29_N_3_O_2_	10.69	368.2332 (− 0.05)	1.3 × 10^6*^	1.0 × 10^5^	4.5 × 10^6^	3.5 × 10^7^	2.4 × 10^6^	9.4 × 10^6^
C14	Oxidative deamination + ω-Carboxylation	C_18_H_18_N_2_O_3_	11.69	311.1389 (− 0.42)	2.3 × 10^7^	ND	2.4 × 10^7^	2.0 × 10^7^	3.1 × 10^7^	3.2 × 10^7^
C15	Oxidative deamination + Hydroxylation (*N*-ethyl)	C_18_H_20_N_2_O_3_	12.04	313.1544 (0.99)	5.5 × 10^6^	4.8 × 10^5^	ND	ND	ND	ND
C16	*N*-Deethylation + O –2H (*N*-alkyl)	C_20_H_23_N_3_O_2_	12.42	338.1860 (− 0.90)	5.6 × 10^7^	ND	ND	ND	ND	ND
C17	Oxidative deamination	C_18_H_20_N_2_O_2_	12.98	297.1597 (− 0.18)	2.6 × 10^7^	ND	1.2 × 10^7^	4.3 × 10^7^	7.2 × 10^5^	6.8 × 10^6^

Mass tolerance, 5 ppm; *ND* not detected

^*^Found retrospectively in incubates after identification in authentic samples

Metabolites were searched in blood (case #5) and urine (cases #3 and #4) samples from etodesnitazene-positive cases (Table 4). Out of 17 metabolites identified in incubates, 15 were detected in either blood or urine: C1, C5, C9, C10, C14, C16, and C17 were not detected in blood; C15 and C16 were not detected in urine, hydrolyzed or not. Five additional hydroxylated metabolites were identified in urine (Ca to Ce by ascending retention time), among which 2 were also identified in blood (Cc and Ce) with low intensity. All 5 additional metabolites were retrospectively detected in incubates, but with a signal intensity below the established threshold. *N*-Deethyl-metonitazene (C12) *O*-deethyl-etodesnitazene (C6), *N*-deethyl-*O*-demethyl-metonitazene (C3), and etodesnitazene were preponderant in blood; and *O*-deethyl-etodesnitazene (C6), *N*-deethyl-O-demethyl-metonitazene (C3), and parent were major in both hydrolyzed urine samples.

**Table 4:** Metabolic transformation, elemental composition, retention time (RT), accurate mass of molecular ion, deviation from theoretical accurate mass, and liquid chromatography-high-resolution mass spectrometry peak area of metodesnitazene and metabolites in positive-ionization mode after 3-h Incubation with human hepatocytes

ID	Biotransformation	Elemental composition	RT, min	*m/z* (Δppm)	Peak area in 3-h incubates
D1	*N*-Deethylation + *O*-Demethylation	C_16_H_17_N_3_O	2.09	296.1756 (1.35)	1.8 × 10^8^
D2	*O*-Demethylation + *O*-Glucuronidation	C_26_H_33_N_3_O_7_	2.47	500.2392 (0.15)	3,0 × 10^6^
D3	*O*-Demethylation	C_20_H_25_N_3_O	4.44	324.2071 (0.18)	1.9 × 10^8^
D4	*N*-Deethylation + Hydroxylation (imidazole)	C_19_H_23_N_3_O_2_	4.63	326.1863 (− 0.01)	2.8 × 10^7^
D5	*N*-Deethylation + *N*-Deethylation	C_17_H_19_N_3_O	4.85	282.1601 (0.04)	3.2 × 10^7^
D6	*N*-Deethylation	C_19_H_23_N_3_O	6.18	310.1911 (− 0.9)	5.9 × 10^7^
D7	Hydroxylation (imidazole)	C_21_H_27_N_3_O_2_	6.35	354.2175 (− 0.3)	4.1 × 10^7^
Metodesnitazene		C_21_H_27_N_3_O	8.17	338.2222 (1.45)	5.3 × 10^8^
D8	*N*-Deethylation + Ketone formation (*N*-alkyl)	C_19_H_21_N_3_O_2_	10.46	324.1705 (− 0.5)	2.5 × 10^7^
D9	Oxidative deamination	C_17_H_18_N_2_O_2_	10.93	283.1440 (− 0.4)	3.9 × 10^7^
D10	*N*-Deethylation + *N*-Oxidation (*N*-alkyl)	C_19_H_23_N_3_O_2_	13.04	326.1863 (− 0.01)	8.5 × 10^6^

Mass tolerance, 5 ppm

## Discussion

### In vitro versus authentic samples and comparison between analogues

**Fig. 5:**
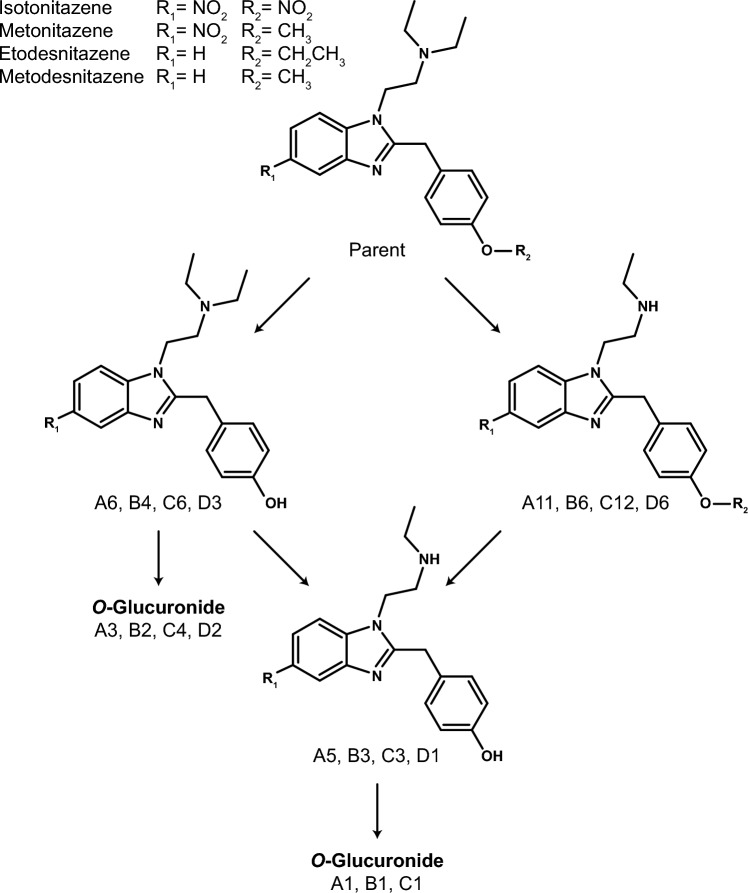
Isotonitazene, metonitazene, etodesnitazene, and metodesnitazene metabolic fate in humans (major metabolites). Gluc, glucuronide

Good correlation was found between metonitazene and etodesnitazene incubations versus authentic positive blood and urine samples as most in vitro metabolites were found in the samples, and major metabolites were the same. The suggested metabolic fate of isotonitazene, metonitazene, etodesnitazene, and metodesnitazene in humans is displayed in Fig. 5. Isotonitazene in vitro results and metonitazene in vitro and postmortem blood and urine results were consistent with previously reported findings from postmortem casework (Krotulski et al. 2020, 2021). However, *N*-deethyl-*O*-demethyl-metonitazene was the principal metonitazene metabolite in the present urine sample but was not reported in the metonitazene-positive case series, although the authors identified *O*-demethyl- and *N*-deethyl-metonitazene in urine samples (Krotulski et al. 2021) and *N*-deethyl-*O*-isopropyl-isotonitazene was found to be a major isotonitazene metabolite in urine (Krotulski et al. 2020); however it is not clear whether the metabolite was searched or not, as the methodology for metabolite identification was not fully described. Etodesnitazene in vitro and postmortem blood and urine results were also consistent with a positive urine in a clinical case (Verougstraete et al. 2023), but they somewhat differ from findings in rat serum and urine, in which hydroxylation and *N*-oxidation were more abundant, which might be explained by inter-species differences (Grigoryev et al. 2023). *O*-glucuronidation following *O*-dealkylation was a major metabolic transformation for both metonitazene and etodesnitazene in the present study, but phase II metabolites were not searched in previous reports. Overall, these results indicate that incubation with human hepatocyte is a suitable predictive model for the metabolism of nitazenes in humans. In a previous report, human hepatocyte incubations also provided good results compared to in vivo for the prediction of the metabolism of etonitazepipne, an *N*-pyrrolidino analogue (Berardinelli D et al. 2024). It is important to highlight however, that, although their intensity was not important in blood and urine, nitro-reduced metabolites were detected with a much lower intensity in metonitazene incubates compared to authentic samples, potentially indicating extra-hepatic metabolism. Contrary to our observations, Krotulski et al. found that 5-amino-metonitazene was the main metabolite in the blood of all 14 cases in which metonitazene metabolites were detected (Krotulski et al. 2021). The authors prepared the samples with a liquid–liquid extraction, which might have affected the relative recovery of the metabolites, but, conversely, the protein precipitation of the present study might have affected the relative matrix effect of the metabolites. Other potential reasons to this discrepancy might be due to the instability of the metabolite, a different postmortem redistribution, or interindividual genetic variations. Further investigation with a purified reference standard of the metabolite is necessary to further understand its significance.

Interestingly, consistent with previously published reports on isotonitazene and metonitazene results from forensic casework (Krotulski et al. 2020, 2021), although *N*-deethyl-metonitazene was major in blood, it was not in urine. Contrarily, *O*-demethyl metabolites, alone and/or in combination with *N*-deethylation, were minor in blood, but were predominant in urine with, but also without β-glucuronidase hydrolysis. *N*-Deethyl-etodesnitazene also was relatively more intense in blood than in urine, although to a lower extent. A lower polarity of *N*-deethyl metabolites compared to *O*-demethyl metabolites for these analogues, reflected in the retention time difference under our analytical conditions, might explain this discrepancy, *O*-dealkyl metabolites undergoing faster urinary elimination. Another explanation might be the elimination of *N*-deethyl metabolites through another route. Nevertheless, the high intensity of *N*-deethyl-metonitazene (B6) and *N*-deethyl-etodesnitazene (C12) in blood is a critical aspect of the metabolism of these two synthetic opioids as they likely are also active and potent. Indeed, *N*-deethyl-isotonitazene proved much more potent than its parent, isotonitazene, in in vitro experiments evaluating MOR activation (Vandeputte et al. 2021), which may well translate to close structural analogues.

Another important observation is the intensity of metonitazene and etodesnitazene in authentic samples, contrasting the parent’s signal being irrelevant in incubates. In metonitazene-positive blood and urine samples, metonitazene signal was more intense than that of its metabolites. In etodesnitazene-positive blood, the parent’s signal was more intense than that of its metabolites, but, although intense, was not predominant in urine; etodesnitazene was the fifth most intense biomarker in case #3 and the third most intense in case #4, behind *N*- and *O*-deethyl metabolites. The time of death after intake might be a reason for this observation, but etodesnitazene might need to undergo further metabolization before urinary elimination, in contrast with metonitazene. Additionally, *O*-dealkylation seemed more frequent in etodesnitazene and metodesnitazene compared to isotonitazene and metonitazene in vitro and in postmortem samples, pointing towards the length and tridimensional conformation of the alkoxy chain being key to metabolic enzyme docking. More data on the pharmacokinetics of the metabolites with purified standards is necessary to fully understand these differences.

### Biomarkers of consumption

The major metabolites identified in human hepatocyte incubations and authentic samples are displayed in Fig. 5.

In urine, *O*-dealkyl and *N*-deethyl-*O*-dealkyl metabolites and glucuronides were predominant. Isotonitazene and metonitazene *O*-dealkyl metabolites were the same molecules and are also likely produced through the metabolization of structural analogues such as etonitazene, protonitazene, or butonitazene, whose only difference from isotonitazene and metonitazene is the composition of the alkoxy side chain. Similarly, etodesnitazene and metodesnitazene *O*-dealkyl metabolites were also the same molecules and might be produced through isotodesnitazene or protodesnitazene metabolization. Therefore, considering the intensity of metonitazene and etodesnitazene in postmortem samples, the parent drug should be detected to attempt to prove consumption, along with *O*-dealkyl and *N*-deethyl-*O*-dealkyl metabolite biomarkers after glucuronide hydrolysis to increase the detection capabilities of phase I metabolites. Alternatively, considering that hydrolysis conditions in routine toxicological screenings are not always suitable and can rarely be optimized in targeted analytical methods in the absence of reference standards, the phase II conjugates of *O*-dealkyl and *N*-deethyl-*O*-dealkyl metabolites are suitable additional targets for proving consumption without glucuronide hydrolysis in urine.

In blood, the *N*-deethyl metabolite was preponderant for metonitazene and etodesnitazene, and *O*-deethyl-etodesnitazene and *N*,*O*-dideethyl-etodesnitazene were also major in the etodesnitazene-positive sample. As for *O*-dealkyl metabolites, *N*-deethyl metabolites also might not be specific: *N*-deethyl-isotonitazene, in addition to being more potent than isotonitazene (Vandeputte et al. 2021), has been also used for recreational purposes (CFSRE 2024). Therefore, considering the intensity of metonitazene and etodesnitazene in postmortem samples, the parent drug should be detected to document consumption, along with *N*-deethyl, *O*-dealkyl, and *N*-deethyl-*O*-dealkyl metabolite biomarkers. According to previous reports (Krotulski et al. 2020, 2021), 5-amino metabolites might also be suitable alternative targets in blood for proving exposure to nitazenes with a nitro group in position 5.

## Conclusions

NPS metabolites of new compounds are difficult to predict, as minor structural modifications may substantially impact their metabolism (Di Trana et al. 2021; Brunetti et al. 2023). Therefore, metabolite identification in humans is necessary to identify specific metabolite biomarkers of NPS consumption (Carlier et al. 2018, 2020, 2022). The present study explored the human metabolism of four nitazene analogues, isotonitazene, metonitazene, etodesnitazene, and metodesnitazene. The identification of these synthetic opioids’ metabolites is especially important considering that several metabolites proved to be highly potent MOR agonists in vitro (Vandeputte et al. 2021). Specific metabolite biomarkers were found to help analytical toxicologists’ documentation of clinical and forensic opioid intoxication cases and enable further pharmacokinetic studies. We suggest the parent, and *O*-dealkyl and *N*-deethyl-*O*-dealkyl metabolites as biomarkers of the four analogues in urine after glucuronide hydrolysis; and the parent, and *N*-deethyl, *O*-dealkyl, and *N*-deethyl-*O*-dealkyl metabolites as biomarkers of exposure in blood. Considering the correlation between in vitro and postmortem samples results, human hepatocyte incubation proved a suitable model for the prediction of benzimidazole opioids’ human metabolism.

### Supplementary Information

Below is the link to the electronic supplementary material.

Supplementary file1 (PDF 301 KB)

Supplementary file2 (PDF 251 KB)

Supplementary file3 (PDF 201 KB)

## Data Availability

Derived data supporting the findings of this study are available from the corresponding author upon request.
