# Supplementary material for: Human metabolism of four synthetic benzimidazole opioids: isotonitazene, metonitazene, etodesnitazene, and metodesnitazene
Source: Arch Toxicol. 2024 Apr 6;98(7):2101–16. doi: 10.1007/s00204-024-03735-0 (PMC11169013; doi:10.1007/s00204-024-03735-0)
Supplement: Supplementary file 1 — Supplementary file1 (PDF 301 KB) [file 204_2024_3735_MOESM1_ESM.pdf]

Table S1-1. Isotonitazene putative metabolites predicted with GLORYx freeware and their prediction score (adjusted score for second-generation metabolites)

Isotonitazene

**ID**	**Transformation**	**Elemental composition**	**Score**	**Simplified molecular-input line-entry system (SMILES)**	**Comment**
pA1	*N*-Deethylation	$C_{21}H_{26}N_4O_3$	78.0%	CC(C)Oc1ccc(cc1)Cc1nc2cc(ccc2n1CCNCC)[N+]([O-])=O	= pA2-6
pA1-1	+ *O*-Deisopropylation	$C_{18}H_{20}N_4O_3$	49.1%	[O-][N+](=O)c1cc2nc(Cc3ccc(O)cc3)n(CCNCC)c2cc1	= pA4-3, pB1-1, pB4-3
pA1-2	+ Hydroxylation (*O*-isopropyl)	$C_{21}H_{26}N_4O_4$	49.1%	CC(C)(O)Oc1ccc(cc1)Cc1nc2cc(ccc2n1CCNCC)[N+]([O-])=O	= pA5-1
pA1-3	+ *N*-Deethylation	$C_{19}H_{22}N_4O_3$	48.4%	CC(C)Oc1ccc(cc1)Cc1nc2cc(ccc2n1CCN)[N+]([O-])=O	
pA1-4	+ Deamination to alcohol	$C_{19}H_{21}N_3O_4$	48.4%	CC(C)Oc1ccc(cc1)Cc1nc2cc(ccc2n1CCO)[N+]([O-])=O	= pA2-14, pA7
pA1-5	+ Hydroxylation (*N*-ethyl)	$C_{21}H_{26}N_4O_4$	48.4%	CC(C)Oc1ccc(cc1)Cc1nc2cc(ccc2n1CCNC(C)O)[N+]([O-])=O	= pA2-11
pA1-6	+ *N*-Oxidation (alkyl)	$C_{21}H_{26}N_4O_4$	48.4%	CC(C)Oc1ccc(cc1)Cc1nc2cc(ccc2n1CCN(O)CC)[N+]([O-])=O	= pA3-6
pA1-7	+ *O*-Glucuronidation (nitro)	$C_{27}H_{35}N_4O_9^+$	45.2%	O=[N+](OC1OC(C(O)C(O)C1O)C(=O)O)c1ccc2n(CCNCC)c(Cc3ccc(OC(C)C)cc3)nc2c1	Charged
pA1-8	+ Hydroxylation (*O*-isopropyl)	$C_{21}H_{26}N_4O_4$	27.3%	CC(CO)Oc1ccc(cc1)Cc1nc2cc(ccc2n1CCNCC)[N+]([O-])=O	
pA2	Hydroxylation (*N*-ethyl)	$C_{23}H_{30}N_4O_4$	78.0%	CC(C)Oc1ccc(cc1)Cc1nc2cc(ccc2n1CCN(CC)C(C)O)[N+]([O-])=O	
pA2-1	+ *O*-Sulfation (hydroxyl)	$C_{23}H_{30}N_4O_7S$	74.1%	CC(C)Oc1ccc(cc1)Cc1nc2cc(ccc2n1CCN(CC)C(C)OS(=O)(=O)O)[N+]([O-])=O	
pA2-2	+ *O*-Deisopropylation	$C_{20}H_{24}N_4O_4$	49.1%	[O-][N+](=O)c1cc2nc(Cc3ccc(O)cc3)n(CCN(CC)C(C)O)c2cc1	= pA4-4, pB2-2, pB4-4
pA2-3	+ Hydroxylation (*O*-isopropyl)	$C_{23}H_{30}N_4O_5$	49.1%	CC(C)(O)Oc1ccc(cc1)Cc1nc2cc(ccc2n1CCN(CC)C(C)O)[N+]([O-])=O	= pA5-2
pA2-4	+ *O*-Glucuronidation (hydroxyl)	$C_{29}H_{38}N_4O_{10}$	45.2%	CC(C)Oc1ccc(cc1)Cc1nc2cc(ccc2n1CCN(CC)C(C)OC1OC(C(O)C(O)C1O)C(=O)O)[N+]([O-])=O	
pA2-5	+ *O*-Glucuronidation (nitro)	$C_{29}H_{39}N_4O_{10}^+$	45.2%	O=[N+](OC1OC(C(O)C(O)C1O)C(=O)O)c1ccc2n(CCN(CC)C(C)O)c(Cc3ccc(OC(C)C)cc3)nc2c1	Charged
pA2-6	+ *N*-Deethylation	$C_{21}H_{26}N_4O_3$	39.0%	CC(C)Oc1ccc(cc1)Cc1nc2cc(ccc2n1CCNCC)[N+]([O-])=O	= pA1
pA2-7	+ Dehydrogenation to ketone (hydroxyl)	$C_{23}H_{28}N_4O_4$	39.0%	CC(C)Oc1ccc(cc1)Cc1nc2cc(ccc2n1CCN(CC)C(C)=O)[N+]([O-])=O	
pA2-8	+ Hydroxylation (*N*-ethyl)	$C_{23}H_{30}N_4O_5$	39.0%	CC(C)Oc1ccc(cc1)Cc1nc2cc(ccc2n1CCN(CC)C(C)(O)O)[N+]([O-])=O	
pA2-9	+ *N*-Oxidation (alkyl)	$C_{23}H_{30}N_4O_5$	39.0%	CC(C)Oc1ccc(cc1)Cc1nc2cc(ccc2n1CC[N+]([O-])(CC)C(C)O)[N+]([O-])=O	= pA3-7
pA2-10	+ Hydroxylation (*O*-isopropyl)	$C_{23}H_{30}N_4O_5$	27.3%	CC(CO)Oc1ccc(cc1)Cc1nc2cc(ccc2n1CCN(CC)C(C)O)[N+]([O-])=O	
pA2-11	+ *N*-Deethylation	$C_{21}H_{26}N_4O_4$	23.4%	CC(C)Oc1ccc(cc1)Cc1nc2cc(ccc2n1CCNC(C)O)[N+]([O-])=O	= pA1-5
pA2-12	+ Hydroxylation (*N*-ethyl)	$C_{23}H_{30}N_4O_5$	23.4%	CC(C)Oc1ccc(cc1)Cc1nc2cc(ccc2n1CCN(C(C)O)C(C)O)[N+]([O-])=O	
pA2-13	+ Deamination to aldehyde	$C_{19}H_{19}N_3O_4$	21.1%	CC(C)Oc1ccc(cc1)Cc1nc2cc(ccc2n1CC=O)[N+]([O-])=O	= pA6
pA2-14	+ Deamination to alcohol	$C_{19}H_{21}N_3O_4$	21.1%	CC(C)Oc1ccc(cc1)Cc1nc2cc(ccc2n1CCO)[N+]([O-])=O	= pA1-4, pA7
pA3	*N*-Oxidation (alkyl)	$C_{23}H_{30}N_4O_4$	78.0%	CC(C)Oc1ccc(cc1)Cc1nc2cc(ccc2n1CC[N+]([O-])(CC)CC)[N+]([O-])=O	
pA3-1	+ *O*-Deisopropylation	$C_{20}H_{24}N_4O_4$	49.1%	[O-][N+](=O)c1cc2nc(Cc3ccc(O)cc3)n(CC[N+]([O-])(CC)CC)c2cc1	= pA4-5, pB3-1, pB4-5
pA3-2	+ Hydroxylation (*O*-isopropyl)	$C_{23}H_{30}N_4O_5$	49.1%	CC(C)(O)Oc1ccc(cc1)Cc1nc2cc(ccc2n1CC[N+]([O-])(CC)CC)[N+]([O-])=O	= pA5-3
pA3-3	+ *O*-Glucuronidation (*N*-oxide)	$C_{29}H_{39}N_4O_{10}^+$	47.6%	CC(C)Oc1ccc(cc1)Cc1nc2cc(ccc2n1CC[N+](OC1OC(C(O)C(O)C1O)C(=O)O)(CC)CC)[N+]([O-])=O	Charged
pA3-4	+ *O*-Glucuronidation (nitro)	$C_{29}H_{39}N_4O_{10}^+$	45.2%	O=[N+](OC1OC(C(O)C(O)C1O)C(=O)O)c1ccc2n(CC[N+]([O-])(CC)CC)c(Cc3ccc(OC(C)C)cc3)nc2c1	Charged
pA3-5	+ Hydroxylation (*O*-isopropyl)	$C_{23}H_{30}N_4O_5$	27.3%	CC(CO)Oc1ccc(cc1)Cc1nc2cc(ccc2n1CC[N+]([O-])(CC)CC)[N+]([O-])=O	
pA3-6	+ *N*-Deethylation	$C_{21}H_{26}N_4O_4$	24.2%	CC(C)Oc1ccc(cc1)Cc1nc2cc(ccc2n1CCN(O)CC)[N+]([O-])=O	= pA1-6
pA3-7	+ Hydroxylation (*N*-ethyl)	$C_{23}H_{30}N_4O_5$	24.2%	CC(C)Oc1ccc(cc1)Cc1nc2cc(ccc2n1CC[N+]([O-])(CC)C(C)O)[N+]([O-])=O	= pA2-9
pA4	*O*-Deisopropylation	$C_{20}H_{24}N_4O_3$	63.0%	[O-][N+](=O)c1cc2nc(Cc3ccc(O)cc3)n(CCN(CC)CC)c2cc1	= pB4, pB5-5
pA4-1	+ *O*-Glucuronidation (hydroxyl)	$C_{26}H_{32}N_4O_9$	61.7%	[O-][N+](=O)c1cc2nc(Cc3ccc(cc3)OC3OC(C(O)C(O)C3O)C(=O)O)n(CCN(CC)CC)c2cc1	= pB4-1
pA4-2	+ *O*-Sulfation (hydroxyl)	$C_{20}H_{24}N_4O_6S$	59.9%	O=S(=O)(O)Oc1ccc(cc1)Cc1nc2cc(ccc2n1CCN(CC)CC)[N+]([O-])=O	= pB4-2
pA4-3	+ *N*-Deethylation	$C_{18}H_{20}N_4O_3$	49.1%	[O-][N+](=O)c1cc2nc(Cc3ccc(O)cc3)n(CCNCC)c2cc1	= pA1-1, pB1-1, pB4-3
pA4-4	+ Hydroxylation (*N*-ethyl)	$C_{20}H_{24}N_4O_4$	49.1%	[O-][N+](=O)c1cc2nc(Cc3ccc(O)cc3)n(CCN(CC)C(C)O)c2cc1	= pA2-2, pB2-2, pB4-4
pA4-5	+ *N*-Oxidation (alkyl)	$C_{20}H_{24}N_4O_4$	49.1%	[O-][N+](=O)c1cc2nc(Cc3ccc(O)cc3)n(CC[N+]([O-])(CC)CC)c2cc1	= pA3-1, pB3-1, pB4-5
pA4-6	+ *O*-Glucuronidation (nitro)	$C_{26}H_{33}N_4O_9^+$	36.5%	O=[N+](OC1OC(C(O)C(O)C1O)C(=O)O)c1ccc2n(CCN(CC)CC)c(Cc3ccc(O)cc3)nc2c1	= pB6-1; Charged
pA4-7	+ Hydroxylation (phenyl)	$C_{20}H_{24}N_4O_4$	29.0%	[O-][N+](=O)c1cc2nc(Cc3ccc(O)c(O)c3)n(CCN(CC)CC)c2cc1	= pB4-6
pA4-8	+ Deamination to aldehyde	$C_{16}H_{13}N_3O_4$	22.7%	[O-][N+](=O)c1cc2nc(Cc3ccc(O)cc3)n(CC=O)c2cc1	= pB4-7
pA4-9	+ Deamination to alcohol	$C_{16}H_{15}N_3O_4$	22.7%	[O-][N+](=O)c1cc2nc(Cc3ccc(O)cc3)n(CCO)c2cc1	= pB4-8
pA5	Hydroxylation (*O*-isopropyl)	$C_{23}H_{30}N_4O_4$	63.0%	CC(C)(O)Oc1ccc(cc1)Cc1nc2cc(ccc2n1CCN(CC)CC)[N+]([O-])=O	
pA5-1	+ *N*-Deethylation	$C_{21}H_{26}N_4O_4$	47.9%	CC(C)(O)Oc1ccc(cc1)Cc1nc2cc(ccc2n1CCNCC)[N+]([O-])=O	= pA1-2
pA5-2	+ Hydroxylation (*N*-ethyl)	$C_{23}H_{30}N_4O_5$	47.9%	CC(C)(O)Oc1ccc(cc1)Cc1nc2cc(ccc2n1CCN(CC)C(C)O)[N+]([O-])=O	= pA2-3
pA5-3	+ *N*-Oxidation (alkyl)	$C_{23}H_{30}N_4O_5$	47.9%	CC(C)(O)Oc1ccc(cc1)Cc1nc2cc(ccc2n1CC[N+]([O-])(CC)CC)[N+]([O-])=O	= pA3-2
pA5-4	+ *O*-Sulfation (hydroxyl)	$C_{23}H_{30}N_4O_7S$	47.9%	O=S(=O)(O)OC(C)(C)Oc1ccc(cc1)Cc1nc2cc(ccc2n1CCN(CC)CC)[N+]([O-])=O	
pA5-5	+ *O*-Glucuronidation (nitro)	$C_{29}H_{39}N_4O_{10}^+$	36.5%	O=[N+](OC1OC(C(O)C(O)C1O)C(=O)O)c1ccc2n(CCN(CC)CC)c(Cc3ccc(OC(C)(C)O)cc3)nc2c1	Charged
pA5-6	+ Deamination to aldehyde	$C_{19}H_{19}N_3O_5$	23.9%	CC(C)(O)Oc1ccc(cc1)Cc1nc2cc(ccc2n1CC=O)[N+]([O-])=O	
pA5-7	+ Deamination to alcohol	$C_{19}H_{21}N_3O_5$	23.9%	CC(C)(O)Oc1ccc(cc1)Cc1nc2cc(ccc2n1CCO)[N+]([O-])=O	
pA6	Deamination to aldehyde	$C_{19}H_{19}N_3O_4$	38.0%	CC(C)Oc1ccc(cc1)Cc1nc2cc(ccc2n1CC=O)[N+]([O-])=O	= pA2-13
pA7	Deamination to alcohol	$C_{19}H_{21}N_3O_4$	38.0%	CC(C)Oc1ccc(cc1)Cc1nc2cc(ccc2n1CCO)[N+]([O-])=O	= pA1-4, pA2-14
pA8	Hydroxylation (*O*-isopropyl)	$C_{23}H_{30}N_4O_4$	35.0%	CC(CO)Oc1ccc(cc1)Cc1nc2cc(ccc2n1CCN(CC)CC)[N+]([O-])=O	
pA9	Hydroxylation (*N*-ethyl)	$C_{23}H_{30}N_4O_4$	23.0%	CC(C)Oc1ccc(cc1)Cc1nc2cc(ccc2n1CCN(CC)CCO)[N+]([O-])=O	
pA10	Hydroxylation (methyl linker)	$C_{23}H_{30}N_4O_4$	20.0%	CC(C)Oc1ccc(cc1)C(O)c1nc2cc(ccc2n1CCN(CC)CC)[N+]([O-])=O	

Table S1-2. Metonitazene putative metabolites predicted with GLORYx freeware and their prediction score (adjusted score for second-generation metabolites)

Metonitazene

ID	Transformation	Elemental composition	Score	Simplified molecular-input line-entry system (SMILES)	Comment
pB1	*N*-Deethylation	$C_{19}H_{22}N_4O_3$	71.0%	[O-][N+](=O)c1cc2nc(Cc3ccc(OC)cc3)n(CCNCC)c2cc1	= pB2-5
pB1-1	+ *O*-Demethylation	$C_{18}H_{20}N_4O_3$	45.4%	[O-][N+](=O)c1cc2nc(Cc3ccc(O)cc3)n(CCNCC)c2cc1	= pA1-1, pA4-3, pB4-3
pB1-2	+ Hydroxylation (*O*-methyl)	$C_{19}H_{22}N_4O_4$	45.4%	[O-][N+](=O)c1cc2nc(Cc3ccc(OCO)cc3)n(CCNCC)c2cc1	= pB5-1
pB1-3	+ *N*-Deethylation	$C_{17}H_{18}N_4O_3$	43.3%	[O-][N+](=O)c1cc2nc(Cc3ccc(OC)cc3)n(CCN)c2cc1	
pB1-4	+ Deamination to alcohol	$C_{17}H_{17}N_3O_4$	43.3%	[O-][N+](=O)c1cc2nc(Cc3ccc(OC)cc3)n(CCO)c2cc1	= pB8
pB1-5	+ Hydroxylation (*N*-ethyl)	$C_{19}H_{22}N_4O_4$	43.3%	[O-][N+](=O)c1cc2nc(Cc3ccc(OC)cc3)n(CCNC(C)O)c2cc1	= pB2-9
pB1-6	+ *N*-Oxidation (alkyl)	$C_{19}H_{22}N_4O_4$	43.3%	[O-][N+](=O)c1cc2nc(Cc3ccc(OC)cc3)n(CCN(O)CC)c2cc1	
pB2	Hydroxylation (*N*-ethyl)	$C_{21}H_{26}N_4O_4$	71.0%	[O-][N+](=O)c1cc2nc(Cc3ccc(OC)cc3)n(CCN(CC)C(C)O)c2cc1	
pB2-1	+ *O*-Sulfation (hydroxyl)	$C_{21}H_{26}N_4O_7S$	68.2%	[O-][N+](=O)c1cc2nc(Cc3ccc(OC)cc3)n(CCN(CC)C(C)OS(=O)(=O)O)c2cc1	
pB2-2	+ *O*-Demethylation	$C_{20}H_{24}N_4O_4$	45.4%	[O-][N+](=O)c1cc2nc(Cc3ccc(O)cc3)n(CCN(CC)C(C)O)c2cc1	= pA2-2, pA4-4, pB4-4
pB2-3	+ Hydroxylation (*O*-methyl)	$C_{21}H_{26}N_4O_5$	45.4%	[O-][N+](=O)c1cc2nc(Cc3ccc(OCO)cc3)n(CCN(CC)C(C)O)c2cc1	= pB5-2
pB2-4	+ *O*-Glucuronidation (hydroxyl)	$C_{27}H_{34}N_4O_{10}$	41.9%	[O-][N+](=O)c1cc2nc(Cc3ccc(OC)cc3)n(CCN(CC)C(C)OC3OC(C(O)C(O)C3O)C(=O)O)c2cc1	
pB2-5	+ *N*-Deethylation	$C_{19}H_{22}N_4O_3$	36.9%	[O-][N+](=O)c1cc2nc(Cc3ccc(OC)cc3)n(CCNCC)c2cc1	= pB1
pB2-6	+ Dehydrogenation to ketone (hydroxyl)	$C_{21}H_{24}N_4O_4$	36.9%	CC(=O)N(CC)CCn1c2ccc(cc2nc1Cc1ccc(OC)cc1)[N+]([O-])=O	
pB2-7	+ Hydroxylation (*N*-ethyl)	$C_{21}H_{26}N_4O_5$	36.9%	[O-][N+](=O)c1cc2nc(Cc3ccc(OC)cc3)n(CCN(CC)C(C)(O)O)c2cc1	
pB2-8	+ *N*-Oxidation (alkyl)	$C_{21}H_{26}N_4O_5$	36.9%	[O-][N+](=O)c1cc2nc(Cc3ccc(OC)cc3)n(CC[N+]([O-])(CC)C(C)O)c2cc1	
pB2-9	+ *N*-Deethylation	$C_{19}H_{22}N_4O_4$	22.0%	[O-][N+](=O)c1cc2nc(Cc3ccc(OC)cc3)n(CCNC(C)O)c2cc1	= pB1-5
pB2-10	+ Hydroxylation (*N*-ethyl)	$C_{21}H_{26}N_4O_5$	22.0%	CC(O)N(CCn1c2ccc(cc2nc1Cc1ccc(OC)cc1)[N+]([O-])=O)C(C)O	
pB3	*N*-Oxidation (alkyl)	$C_{21}H_{26}N_4O_4$	71.0%	[O-][N+](=O)c1cc2nc(Cc3ccc(OC)cc3)n(CC[N+]([O-])(CC)CC)c2cc1	
pB3-1	+ *O*-Demethylation	$C_{20}H_{24}N_4O_4$	44.0%	[O-][N+](=O)c1cc2nc(Cc3ccc(O)cc3)n(CC[N+]([O-])(CC)CC)c2cc1	= pA3-1, pA4-5, pB4-5
pB3-2	+ Hydroxylation (*O*-methyl)	$C_{21}H_{26}N_4O_5$	44.0%	[O-][N+](=O)c1cc2nc(Cc3ccc(OCO)cc3)n(CC[N+]([O-])(CC)CC)c2cc1	= pB5-3
pB4	*O*-Demethylation	$C_{20}H_{24}N_4O_3$	64.0%	[O-][N+](=O)c1cc2nc(Cc3ccc(O)cc3)n(CCN(CC)CC)c2cc1	= pA4, pB5-5
pB4-1	+ *O*-Glucuronidation (hydroxyl)	$C_{26}H_{32}N_4O_9$	62.7%	[O-][N+](=O)c1cc2nc(Cc3ccc(cc3)OC3OC(C(O)C(O)C3O)C(=O)O)n(CCN(CC)CC)c2cc1	= pA4-1
pB4-2	+ *O*-Sulfation (hydroxyl)	$C_{20}H_{24}N_4O_6S$	60.8%	O=S(=O)(O)Oc1ccc(cc1)Cc1nc2cc(ccc2n1CCN(CC)CC)[N+]([O-])=O	= pA4-2
pB4-3	+ *N*-Deethylation	$C_{18}H_{20}N_4O_3$	49.9%	[O-][N+](=O)c1cc2nc(Cc3ccc(O)cc3)n(CCNCC)c2cc1	= pA1-1, pA4-3, pB1-1
pB4-4	+ Hydroxylation (*N*-ethyl)	$C_{20}H_{24}N_4O_4$	49.9%	[O-][N+](=O)c1cc2nc(Cc3ccc(O)cc3)n(CCN(CC)C(C)O)c2cc1	= pA2-2, pA4-4, pB2-2
pB4-5	+ *N*-Oxidation (alkyl)	$C_{20}H_{24}N_4O_4$	49.9%	[O-][N+](=O)c1cc2nc(Cc3ccc(O)cc3)n(CC[N+]([O-])(CC)CC)c2cc1	= pA3-1, pA4-5, pB3-1
pB4-6	+ Hydroxylation (phenyl)	$C_{20}H_{24}N_4O_4$	29.4%	[O-][N+](=O)c1cc2nc(Cc3ccc(O)c(O)c3)n(CCN(CC)CC)c2cc1	= pA4-7
pB4-7	+ Deamination to aldehyde	$C_{16}H_{13}N_3O_4$	23.0%	[O-][N+](=O)c1cc2nc(Cc3ccc(O)cc3)n(CC=O)c2cc1	= pA4-8
pB4-8	+ Deamination to alcohol	$C_{16}H_{15}N_3O_4$	23.0%	[O-][N+](=O)c1cc2nc(Cc3ccc(O)cc3)n(CCO)c2cc1	= pA4-9
pB5	Hydroxylation (*O*-methyl)	$C_{21}H_{26}N_4O_4$	64.0%	[O-][N+](=O)c1cc2nc(Cc3ccc(OCO)cc3)n(CCN(CC)CC)c2cc1	
pB5-1	+ *N*-Deethylation	$C_{19}H_{22}N_4O_4$	49.9%	[O-][N+](=O)c1cc2nc(Cc3ccc(OCO)cc3)n(CCNCC)c2cc1	= pB1-2
pB5-2	+ Hydroxylation (*N*-ethyl)	$C_{21}H_{26}N_4O_5$	49.9%	[O-][N+](=O)c1cc2nc(Cc3ccc(OCO)cc3)n(CCN(CC)C(C)O)c2cc1	= pB2-3
pB5-3	+ *N*-Oxidation (alkyl)	$C_{21}H_{26}N_4O_5$	49.9%	[O-][N+](=O)c1cc2nc(Cc3ccc(OCO)cc3)n(CC[N+]([O-])(CC)CC)c2cc1	= pB3-2
pB5-4	+ *O*-Sulfation (hydroxyl)	$C_{21}H_{26}N_4O_7S$	49.3%	O=S(=O)(O)OCOc1ccc(cc1)Cc1nc2cc(ccc2n1CCN(CC)CC)[N+]([O-])=O	
pB5-5	+ *O*-Demethylation	$C_{20}H_{24}N_4O_3$	37.8%	[O-][N+](=O)c1cc2nc(Cc3ccc(O)cc3)n(CCN(CC)CC)c2cc1	= pA4, pB4
pB5-6	+ Hydroxylation (*O*-methyl)	$C_{21}H_{26}N_4O_5$	37.8%	OC(O)Oc1ccc(cc1)Cc1nc2cc(ccc2n1CCN(CC)CC)[N+]([O-])=O	
pB5-7	+ Dehydrogenation to aldehyde (hydroxyl)	$C_{21}H_{24}N_4O_4$	37.8%	[O-][N+](=O)c1cc2nc(Cc3ccc(OC=O)cc3)n(CCN(CC)CC)c2cc1	
pB5-8	+ Deamination to aldehyde	$C_{17}H_{15}N_3O_5$	24.3%	[O-][N+](=O)c1cc2nc(Cc3ccc(OCO)cc3)n(CC=O)c2cc1	
pB5-9	+ Deamination to alcohol	$C_{17}H_{17}N_3O_5$	24.3%	[O-][N+](=O)c1cc2nc(Cc3ccc(OCO)cc3)n(CCO)c2cc1	
pB6	*O*-Glucuronidation (*N*-oxide)	$C_{27}H_{35}N_4O_9^+$	58.0%	O=[N+](OC1OC(C(O)C(O)C1O)C(=O)O)c1ccc2n(CCN(CC)CC)c(Cc3ccc(OC)cc3)nc2c1	Charged
pB6-1	+ *O*-Demethylation	$C_{26}H_{33}N_4O_9^+$	36.5%	O=[N+](OC1OC(C(O)C(O)C1O)C(=O)O)c1ccc2n(CCN(CC)CC)c(Cc3ccc(O)cc3)nc2c1	= pA4-6; Charged
pB6-2	+ Hydroxylation (*O*-methyl)	$C_{27}H_{35}N_4O_{10}^+$	36.5%	O=[N+](OC1OC(C(O)C(O)C1O)C(=O)O)c1ccc2n(CCN(CC)CC)c(Cc3ccc(OCO)cc3)nc2c1	Charged
pB6-3	+ *N*-Deethylation	$C_{25}H_{31}N_4O_9^+$	31.9%	O=[N+](OC1OC(C(O)C(O)C1O)C(=O)O)c1ccc2n(CCNCC)c(Cc3ccc(OC)cc3)nc2c1	Charged
pB6-4	+ Hydroxylation (*N*-ethyl)	$C_{27}H_{35}N_4O_{10}^+$	31.9%	O=[N+](OC1OC(C(O)C(O)C1O)C(=O)O)c1ccc2n(CCN(CC)C(C)O)c(Cc3ccc(OC)cc3)nc2c1	Charged
pB6-5	+ *N*-Oxidation (alkyl)	$C_{27}H_{35}N_4O_{10}^+$	31.9%	O=[N+](OC1OC(C(O)C(O)C1O)C(=O)O)c1ccc2n(CC[N+]([O-])(CC)CC)c(Cc3ccc(OC)cc3)nc2c1	Charged
pB7	Deamination to aldehyde	$C_{17}H_{15}N_3O_4$	37.0%	[O-][N+](=O)c1cc2nc(Cc3ccc(OC)cc3)n(CC=O)c2cc1	
pB8	Deamination to alcohol	$C_{17}H_{17}N_3O_4$	37.0%	[O-][N+](=O)c1cc2nc(Cc3ccc(OC)cc3)n(CCO)c2cc1	= pB1-4
pB9	Hydroxylation (methyl linker)	$C_{21}H_{26}N_4O_4$	24.0%	COc1ccc(cc1)C(O)c1nc2cc(ccc2n1CCN(CC)CC)[N+]([O-])=O	
pB10	Hydroxylation (benzimidazole)	$C_{21}H_{26}N_4O_4$	23.0%	[O-][N+](=O)c1ccc2c(nc(Cc3ccc(OC)cc3)n2CCN(CC)CC)c1O	
pB11	*N*-Oxidation (nitro)	$C_{21}H_{28}N_4O_4$	23.0%	[O-][N+](O)(O)c1cc2nc(Cc3ccc(OC)cc3)n(CCN(CC)CC)c2cc1	

Table S1-3. Etodesnitazene putative metabolites predicted with GLORYx freeware and their prediction score (adjusted score for second-generation metabolites)

Etodesnitazene

ID	Transformation	Elemental composition	Score	Simplified molecular-input line-entry system (SMILES)	Comment
pC1	*N*-Deethylation	$C_{20}H_{25}N_3O$	78.0%	CCOc1ccc(cc1)Cc1nc2ccccc2n1CCNCC	= pC2-5
pC1-1	+ *O*-Deethylation	$C_{18}H_{21}N_3O$	58.5%	Oc1ccc(cc1)Cc1nc2ccccc2n1CCNCC	= pC4-3, pD1-1, pD4-3
pC1-2	+ Hydroxylation (*O*-ethyl)	$C_{20}H_{25}N_3O_2$	58.5%	CC(O)Oc1ccc(cc1)Cc1nc2ccccc2n1CCNCC	= pC5-1
pC1-3	+ *N*-Deethylation	$C_{18}H_{21}N_3O$	49.1%	CCOc1ccc(cc1)Cc1nc2ccccc2n1CCN	
pC1-4	+ Deamination to alcohol	$C_{18}H_{20}N_2O_2$	49.1%	CCOc1ccc(cc1)Cc1nc2ccccc2n1CCO	= pC2-12, pC7
pC1-5	+ Hydroxylation (*N*-ethyl)	$C_{20}H_{25}N_3O_2$	49.1%	CCOc1ccc(cc1)Cc1nc2ccccc2n1CCNC(C)O	= pC2-9
pC1-6	+ *N*-Oxidation (alkyl)	$C_{20}H_{25}N_3O_2$	49.1%	CCOc1ccc(cc1)Cc1nc2ccccc2n1CCN(O)CC	= pC3-3
pC2	Hydroxylation (*N*-ethyl)	$C_{22}H_{29}N_3O_2$	78.0%	CCOc1ccc(cc1)Cc1nc2ccccc2n1CCN(CC)C(C)O	
pC2-1	+ *O*-Sulfation (hydroxyl)	$C_{22}H_{29}N_3O_5S$	74.1%	CCOc1ccc(cc1)Cc1nc2ccccc2n1CCN(CC)C(C)OS(=O)(=O)O	
pC2-2	+ *O*-Deethylation	$C_{20}H_{25}N_3O_2$	59.3%	Oc1ccc(cc1)Cc1nc2ccccc2n1CCN(CC)C(C)O	= pC4-4, pD2-2, pD4-4
pC2-3	+ Hydroxylation (*O*-ethyl)	$C_{22}H_{29}N_3O_3$	59.3%	CC(O)Oc1ccc(cc1)Cc1nc2ccccc2n1CCN(CC)C(C)O	= pC5-2
pC2-4	+ *O*-Glucuronidation (hydroxyl)	$C_{28}H_{37}N_3O_8$	45.2%	CCOc1ccc(cc1)Cc1nc2ccccc2n1CCN(CC)C(C)OC1OC(C(O)C(O)C1O)C(=O)O	
pC2-5	+ *N*-Deethylation	$C_{20}H_{25}N_3O$	39.0%	CCOc1ccc(cc1)Cc1nc2ccccc2n1CCNCC	= pC1
pC2-6	+ Dehydrogenation to ketone (hydroxyl)	$C_{22}H_{27}N_3O_2$	39.0%	CC(=O)N(CC)CCn1c2ccccc2nc1Cc1ccc(OCC)cc1	
pC2-7	+ Hydroxylation (*N*-ethyl)	$C_{22}H_{29}N_3O_3$	39.0%	CCOc1ccc(cc1)Cc1nc2ccccc2n1CCN(CC)C(C)(O)O	
pC2-8	+ *N*-Oxidation (alkyl)	$C_{22}H_{29}N_3O_3$	39.0%	CCOc1ccc(cc1)Cc1nc2ccccc2n1CC[N+]([O-])(CC)C(C)O	= pC3-4
pC2-9	+ *N*-Deethylation	$C_{20}H_{25}N_3O_2$	25.0%	CCOc1ccc(cc1)Cc1nc2ccccc2n1CCNC(C)O	= pC1-5
pC2-10	+ Hydroxylation (*N*-ethyl)	$C_{22}H_{29}N_3O_3$	25.0%	CC(O)N(CCn1c2ccccc2nc1Cc1ccc(OCC)cc1)C(C)O	
pC2-11	+ Deamination to aldehyde	$C_{18}H_{18}N_2O_2$	20.3%	CCOc1ccc(cc1)Cc1nc2ccccc2n1CC=O	= pC3-5, pC6
pC2-12	+ Deamination to alcohol	$C_{18}H_{20}N_2O_2$	20.3%	CCOc1ccc(cc1)Cc1nc2ccccc2n1CCO	= pC1-4, pC7
pC3	*N*-Oxidation (alkyl)	$C_{22}H_{29}N_3O_2$	78.0%	CCOc1ccc(cc1)Cc1nc2ccccc2n1CC[N+]([O-])(CC)CC	
pC3-1	+ *O*-Deethylation	$C_{20}H_{25}N_3O_2$	59.3%	Oc1ccc(cc1)Cc1nc2ccccc2n1CC[N+]([O-])(CC)CC	= pC4-5, pD3-1, pD4-5
pC3-2	+ Hydroxylation (*O*-ethyl)	$C_{22}H_{29}N_3O_3$	59.3%	CC(O)Oc1ccc(cc1)Cc1nc2ccccc2n1CC[N+]([O-])(CC)CC	= pC5-3
pC3-3	+ *N*-Deethylation	$C_{20}H_{25}N_3O_2$	21.1%	CCOc1ccc(cc1)Cc1nc2ccccc2n1CCN(O)CC	= pC1-6
pC3-4	+ Hydroxylation (*N*-ethyl)	$C_{22}H_{29}N_3O_3$	21.1%	CCOc1ccc(cc1)Cc1nc2ccccc2n1CC[N+]([O-])(CC)C(C)O	= pC2-8
pC3-5	+ Deamination to aldehyde	$C_{18}H_{20}N_2O_2$	20.3%	CCOc1ccc(cc1)Cc1nc2ccccc2n1CC=O	= pC2-11, pC6
pC4	*O*-Deethylation	$C_{20}H_{25}N_3O$	76.0%	Oc1ccc(cc1)Cc1nc2ccccc2n1CCN(CC)CC	= pC5-5, pD4, pD5-5
pC4-1	+ *O*-Glucuronidation (hydroxyl)	$C_{26}H_{33}N_3O_7$	74.5%	CCN(CC)CCn1c2ccccc2nc1Cc1ccc(cc1)OC1OC(C(O)C(O)C1O)C(=O)O	= pD4-1
pC4-2	+ *O*-Sulfation (hydroxyl)	$C_{20}H_{25}N_3O_4S$	72.2%	O=S(=O)(O)Oc1ccc(cc1)Cc1nc2ccccc2n1CCN(CC)CC	= pD4-2
pC4-3	+ *N*-Deethylation	$C_{18}H_{21}N_3O$	59.3%	Oc1ccc(cc1)Cc1nc2ccccc2n1CCNCC	= pC1-1, pD1-1, pD4-3
pC4-4	+ Hydroxylation (*N*-ethyl)	$C_{20}H_{25}N_3O_2$	59.3%	Oc1ccc(cc1)Cc1nc2ccccc2n1CCN(CC)C(C)O	= pC2-2, pD2-2, pD4-4
pC4-5	+ *N*-Oxidation (alkyl)	$C_{20}H_{25}N_3O_2$	59.3%	Oc1ccc(cc1)Cc1nc2ccccc2n1CC[N+]([O-])(CC)CC	= pC3-1, pD3-1, pD4-5
pC4-6	+ Hydroxylation (phenyl)	$C_{20}H_{25}N_3O_2$	35.0%	Oc1ccc(cc1O)Cc1nc2ccccc2n1CCN(CC)CC	= pD4-6
pC4-7	+ Deamination to aldehyde	$C_{16}H_{14}N_2O_2$	25.8%	Oc1ccc(cc1)Cc1nc2ccccc2n1CC=O	= pD4-7
pC4-8	+ Deamination to alcohol	$C_{16}H_{16}N_2O_2$	25.8%	Oc1ccc(cc1)Cc1nc2ccccc2n1CCO	= pD4-8
pC5	Hydroxylation (*O*-ethyl)	$C_{22}H_{29}N_3O_2$	76.0%	CC(O)Oc1ccc(cc1)Cc1nc2ccccc2n1CCN(CC)CC	
pC5-1	+ *N*-Deethylation	$C_{20}H_{25}N_3O_2$	59.3%	CC(O)Oc1ccc(cc1)Cc1nc2ccccc2n1CCNCC	= pC1-2
pC5-2	+ Hydroxylation (*N*-ethyl)	$C_{22}H_{29}N_3O_3$	59.3%	CC(O)Oc1ccc(cc1)Cc1nc2ccccc2n1CCN(CC)C(C)O	= pC2-3
pC5-3	+ *N*-Oxidation (alkyl)	$C_{22}H_{29}N_3O_3$	59.3%	CC(O)Oc1ccc(cc1)Cc1nc2ccccc2n1CC[N+]([O-])(CC)CC	= pC3-2
pC5-4	+ *O*-Sulfation (hydroxyl)	$C_{22}H_{29}N_3O_5S$	57.8%	O=S(=O)(O)OC(C)Oc1ccc(cc1)Cc1nc2ccccc2n1CCN(CC)CC	
pC5-5	+ *O*-Deethylation	$C_{20}H_{25}N_3O$	47.9%	Oc1ccc(cc1)Cc1nc2ccccc2n1CCN(CC)CC	= pC4, pD4, pD5-5
pC5-6	+ Hydroxylation (*O*-ethyl)	$C_{22}H_{29}N_3O_3$	47.9%	CC(O)(O)Oc1ccc(cc1)Cc1nc2ccccc2n1CCN(CC)CC	
pC5-7	+ Dehydrogenation to ketone (hydroxyl)	$C_{22}H_{27}N_3O_2$	47.9%	CC(=O)Oc1ccc(cc1)Cc1nc2ccccc2n1CCN(CC)CC	
pC5-8	+ Deamination to aldehyde	$C_{18}H_{18}N_2O_3$	28.1%	CC(O)Oc1ccc(cc1)Cc1nc2ccccc2n1CC=O	
pC5-9	+ Deamination to alcohol	$C_{18}H_{20}N_2O_3$	28.1%	CC(O)Oc1ccc(cc1)Cc1nc2ccccc2n1CCO	
pC6	Deamination to aldehyde	$C_{18}H_{18}N_2O_2$	37.0%	CCOc1ccc(cc1)Cc1nc2ccccc2n1CC=O	= pC2-11, pC3-5
pC7	Deamination to alcohol	$C_{18}H_{20}N_2O_2$	37.0%	CCOc1ccc(cc1)Cc1nc2ccccc2n1CCO	= pC1-4, pC2-12
pC8	Hydroxylation (*N*-ethyl)	$C_{22}H_{29}N_3O_2$	23.0%	CCOc1ccc(cc1)Cc1nc2ccccc2n1CCN(CC)CCO	
pC9	Hydroxylation (methyl linker)	$C_{22}H_{29}N_3O_2$	20.0%	CCOc1ccc(cc1)C(O)c1nc2ccccc2n1CCN(CC)CC	
pC10	Hydroxylation (*O*-ethyl)	$C_{22}H_{29}N_3O_2$	20.0%	OCCOc1ccc(cc1)Cc1nc2ccccc2n1CCN(CC)CC	

Table S1-3. Metodesnitazene putative metabolites predicted with GLORYx freeware and their prediction score (adjusted score for second-generation metabolites)

Metodesnitazene

ID	Transformation	Elemental composition	Score	Simplified molecular-input line-entry system (SMILES)	Comment
pD1	*N*-Deethylation	$C_{19}H_{23}N_3O$	71.0%	COc1ccc(cc1)Cc1nc2ccccc2n1CCNCC	= pD2-5
pD1-1	+ *O*-Demethylation	$C_{18}H_{21}N_3O$	45.4%	Oc1ccc(cc1)Cc1nc2ccccc2n1CCNCC	= pC1-1, pC4-3, pD4-3
pD1-2	+ Hydroxylation (*O*-methyl)	$C_{19}H_{23}N_3O_2$	45.4%	OCOc1ccc(cc1)Cc1nc2ccccc2n1CCNCC	= pD5-1
pD1-3	+ *N*-Deethylation	$C_{17}H_{19}N_3O$	44.0%	COc1ccc(cc1)Cc1nc2ccccc2n1CCN	
pD1-4	+ Deamination to alcohol	$C_{17}H_{18}N_2O_2$	44.0%	COc1ccc(cc1)Cc1nc2ccccc2n1CCO	= pD7
pD1-5	+ Hydroxylation (*N*-ethyl)	$C_{19}H_{23}N_3O_2$	44.0%	COc1ccc(cc1)Cc1nc2ccccc2n1CCNC(C)O	= pD2-9
pD1-6	+ *N*-Oxidation (alkyl)	$C_{19}H_{23}N_3O_2$	44.0%	COc1ccc(cc1)Cc1nc2ccccc2n1CCN(O)CC	
pD2	Hydroxylation (*N*-ethyl)	$C_{21}H_{27}N_3O_2$	71.0%	COc1ccc(cc1)Cc1nc2ccccc2n1CCN(CC)C(C)O	
pD2-1	+ *O*-Sulfation (hydroxyl)	$C_{21}H_{27}N_3O_5S$	68.2%	COc1ccc(cc1)Cc1nc2ccccc2n1CCN(CC)C(C)OS(=O)(=O)O	
pD2-2	+ *O*-Demethylation	$C_{20}H_{25}N_3O_2$	45.4%	Oc1ccc(cc1)Cc1nc2ccccc2n1CCN(CC)C(C)O	= pC2-2, pC4-4, pD4-4
pD2-3	+ Hydroxylation (*O*-methyl)	$C_{21}H_{27}N_3O_3$	45.4%	OCOc1ccc(cc1)Cc1nc2ccccc2n1CCN(CC)C(C)O	= pD5-2
pD2-4	+ *O*-Glucuronidation (hydroxyl)	$C_{27}H_{35}N_3O_8$	41.9%	COc1ccc(cc1)Cc1nc2ccccc2n1CCN(CC)C(C)OC1OC(C(O)C(O)C1O)C(=O)O	
pD2-5	+ *N*-Deethylation	$C_{19}H_{23}N_3O$	36.9%	COc1ccc(cc1)Cc1nc2ccccc2n1CCNCC	= pD1
pD2-6	+ Dehydrogenation to ketone (hydroxyl)	$C_{21}H_{25}N_3O_2$	36.9%	CC(=O)N(CC)CCn1c2ccccc2nc1Cc1ccc(OC)cc1	
pD2-7	+ Hydroxylation (*N*-ethyl)	$C_{21}H_{27}N_3O_3$	36.9%	COc1ccc(cc1)Cc1nc2ccccc2n1CCN(CC)C(C)(O)O	
pD2-8	+ *N*-Oxidation (alkyl)	$C_{21}H_{27}N_3O_3$	36.9%	COc1ccc(cc1)Cc1nc2ccccc2n1CC[N+]([O-])(CC)C(C)O	
pD2-9	+ *N*-Deethylation	$C_{19}H_{23}N_3O_2$	22.0%	COc1ccc(cc1)Cc1nc2ccccc2n1CCNC(C)O	= pD1-5
pD2-10	+ Hydroxylation (*N*-ethyl)	$C_{21}H_{27}N_3O_3$	22.0%	CC(O)N(CCn1c2ccccc2nc1Cc1ccc(OC)cc1)C(C)O	
pD3	*N*-Oxidation (alkyl)	$C_{21}H_{27}N_3O_2$	71.0%	COc1ccc(cc1)Cc1nc2ccccc2n1CC[N+]([O-])(CC)CC	
pD3-1	+ *O*-Demethylation	$C_{20}H_{25}N_3O_2$	44.0%	Oc1ccc(cc1)Cc1nc2ccccc2n1CC[N+]([O-])(CC)CC	= pC3-1, pC4-5, pD4-5
pD3-2	+ Hydroxylation (*O*-methyl)	$C_{21}H_{27}N_3O_3$	44.0%	OCOc1ccc(cc1)Cc1nc2ccccc2n1CC[N+]([O-])(CC)CC	= pD5-3
pD4	*O*-Demethylation	$C_{20}H_{25}N_3O$	64.0%	Oc1ccc(cc1)Cc1nc2ccccc2n1CCN(CC)CC	= pC4, pC5-5, pD5-5
pD4-1	+ *O*-Glucuronidation (hydroxyl)	$C_{26}H_{33}N_3O_7$	62.7%	CCN(CC)CCn1c2ccccc2nc1Cc1ccc(cc1)OC1OC(C(O)C(O)C1O)C(=O)O	= pC4-1
pD4-2	+ *O*-Sulfation (hydroxyl)	$C_{20}H_{25}N_3O_4S$	60.8%	O=S(=O)(O)Oc1ccc(cc1)Cc1nc2ccccc2n1CCN(CC)CC	= pC4-2
pD4-3	+ *N*-Deethylation	$C_{18}H_{21}N_3O$	49.9%	Oc1ccc(cc1)Cc1nc2ccccc2n1CCNCC	= pC1-1, pC4-3, pD1-1
pD4-4	+ Hydroxylation (*N*-ethyl)	$C_{20}H_{25}N_3O_2$	49.9%	Oc1ccc(cc1)Cc1nc2ccccc2n1CCN(CC)C(C)O	= pC2-2, pC4-4, pD2-2
pD4-5	+ *N*-Oxidation (alkyl)	$C_{20}H_{25}N_3O_2$	49.9%	Oc1ccc(cc1)Cc1nc2ccccc2n1CC[N+]([O-])(CC)CC	= pC3-1, pC4-5, pD3-1
pD4-6	+ Hydroxylation (phenyl)	$C_{20}H_{25}N_3O_2$	29.4%	Oc1ccc(cc1O)Cc1nc2ccccc2n1CCN(CC)CC	= pC4-6
pD4-7	+ Deamination to aldehyde	$C_{16}H_{14}N_2O_2$	21.8%	Oc1ccc(cc1)Cc1nc2ccccc2n1CC=O	= pC4-7
pD4-8	+ Deamination to alcohol	$C_{16}H_{16}N_2O_2$	21.8%	Oc1ccc(cc1)Cc1nc2ccccc2n1CCO	= pC4-8
pD5	Hydroxylation (*O*-methyl)	$C_{21}H_{27}N_3O_2$	64.0%	OCOc1ccc(cc1)Cc1nc2ccccc2n1CCN(CC)CC	
pD5-1	+ *N*-Deethylation	$C_{19}H_{23}N_3O_2$	49.9%	OCOc1ccc(cc1)Cc1nc2ccccc2n1CCNCC	= pD1-2
pD5-2	+ Hydroxylation (*N*-ethyl)	$C_{21}H_{27}N_3O_3$	49.9%	OCOc1ccc(cc1)Cc1nc2ccccc2n1CCN(CC)C(C)O	= pD2-3
pD5-3	+ *N*-Oxidation (alkyl)	$C_{21}H_{27}N_3O_3$	49.9%	OCOc1ccc(cc1)Cc1nc2ccccc2n1CC[N+]([O-])(CC)CC	= pD3-2
pD5-4	+ *O*-Sulfation (hydroxyl)	$C_{21}H_{27}N_3O_5S$	49.3%	O=S(=O)(O)OCOc1ccc(cc1)Cc1nc2ccccc2n1CCN(CC)CC	
pD5-5	+ *O*-Demethylation	$C_{20}H_{25}N_3O$	37.8%	Oc1ccc(cc1)Cc1nc2ccccc2n1CCN(CC)CC	= pC4, pC5-5, pD4
pD5-6	+ Hydroxylation (*O*-methyl)	$C_{21}H_{27}N_3O_3$	37.8%	OC(O)Oc1ccc(cc1)Cc1nc2ccccc2n1CCN(CC)CC	
pD5-7	+ Dehydrogenation to aldehyde (hydroxyl)	$C_{21}H_{25}N_3O_2$	37.8%	O=COc1ccc(cc1)Cc1nc2ccccc2n1CCN(CC)CC	
pD5-8	+ Deamination to aldehyde	$C_{17}H_{16}N_2O_3$	23.7%	OCOc1ccc(cc1)Cc1nc2ccccc2n1CC=O	
pD5-9	+ Deamination to alcohol	$C_{17}H_{18}N_2O_3$	23.7%	OCOc1ccc(cc1)Cc1nc2ccccc2n1CCO	
pD6	Deamination to aldehyde	$C_{17}H_{16}N_2O_2$	35.0%	COc1ccc(cc1)Cc1nc2ccccc2n1CC=O	
pD7	Deamination to alcohol	$C_{17}H_{18}N_2O_2$	35.0%	COc1ccc(cc1)Cc1nc2ccccc2n1CCO	= pD1-4
pD8	Hydroxylation (methyl linker)	$C_{21}H_{27}N_3O_2$	24.0%	COc1ccc(cc1)C(O)c1nc2ccccc2n1CCN(CC)CC	
