# Supplementary material for: Human metabolism of four synthetic benzimidazole opioids: isotonitazene, metonitazene, etodesnitazene, and metodesnitazene
Source: Arch Toxicol. 2024 Apr 6;98(7):2101–16. doi: 10.1007/s00204-024-03735-0 (PMC11169013; doi:10.1007/s00204-024-03735-0)
Supplement: Supplementary file 2 — Supplementary file2 (PDF 251 KB) [file 204_2024_3735_MOESM2_ESM.pdf]

Table S2-1. Inclusion list used during liquid chromatography-high-resolution tandem mass spectrometry (LC-HRMS/MS) for isotonitazene metabolite identification

Isotonitazene

**Transformation**	**Elemental composition**	**$[M+H]^+$ *m/z***	**$[M-H]^-$ *m/z***
Parent (Isotonitazene)	$C_{23}H_{30}N_4O_3$	411.2391	409.2245
–3C –6H	$C_{20}H_{24}N_4O_3$	369.1921	367.1776
–2C –4H	$C_{21}H_{26}N_4O_3$	383.2078	381.1932
+2H –2O	$C_{23}H_{32}N_4O$	381.2649	379.2503
–O	$C_{23}H_{30}N_4O_2$	395.2442	393.2296
+2H –O	$C_{23}H_{32}N_4O_2$	397.2598	395.2453
+O	$C_{23}H_{30}N_4O_4$	427.2340	425.2194
+6C +9H +6O	$C_{29}H_{39}N_4O_9$	587.2712	-
+10C +16H +2N +4O +S	$C_{33}H_{46}N_6O_7S$	671.3221	669.3076
+5C +8H +O +S	$C_{28}H_{38}N_4O_4S$	527.2687	525.2541
–5C –10H	$C_{18}H_{20}N_4O_3$	341.1608	339.1463
–4C –8H	$C_{19}H_{22}N_4O_3$	355.1765	353.1619
–4C –9H –N +O	$C_{19}H_{21}N_3O_4$	356.1605	354.1459
–4C –11H –N +O	$C_{19}H_{19}N_3O_4$	354.1448	352.1303
–4C –11H –N +2O	$C_{19}H_{19}N_3O_5$	370.1397	368.1252
+3C +2H +6O	$C_{26}H_{32}N_4O_9$	545.2242	543.2097
–3C –6H +3O +S	$C_{20}H_{24}N_4O_6S$	449.1489	447.1344
+2C –H –N +7O	$C_{25}H_{29}N_3O_{10}$	532.1926	530.1780
–4C –9H –N +4O +S	$C_{19}H_{21}N_3O_7S$	436.1173	434.1027
+2C +4H –O	$C_{25}H_{34}N_4O_2$	423.2755	421.2609
+2H +2O	$C_{23}H_{32}N_4O_5$	445.2445	443.2300
–2H	$C_{23}H_{28}N_4O_3$	409.2234	407.2089
–2H +O	$C_{23}H_{28}N_4O_4$	425.2183	423.2038
–3C –6H +O	$C_{20}H_{24}N_4O_4$	385.1870	383.1725
–7C –15H –N +O	$C_{16}H_{15}N_3O_4$	314.1135	312.0990
–7C –17H –N +O	$C_{16}H_{13}N_3O_4$	312.0979	310.0833
–2C –4H +O	$C_{21}H_{26}N_4O_4$	399.2027	397.1881
+4C +5H +6O	$C_{27}H_{35}N_4O_9$	559.2399	-
+2O	$C_{23}H_{30}N_4O_5$	443.2289	441.2143
+6C +8H +7O	$C_{29}H_{38}N_4O_{10}$	603.2661	601.2515
+4O +S	$C_{23}H_{30}N_4O_7S$	507.1908	505.1762
–4C –9H –N +2O	$C_{19}H_{21}N_3O_5$	372.1554	370.1408

Table S2-2. Inclusion list used during liquid chromatography-high-resolution tandem mass spectrometry (LC-HRMS/MS) for metonitazene metabolite identification

Metonitazene

**Transformation**	**Elemental composition**	**[M+H]$^+$ *m/z***	**[M-H]$^-$ *m/z***
Parent (Metonitazene)	$C_{21}H_{26}N_4O_3$	383.2078	381.1932
–C –2H	$C_{20}H_{24}N_4O_3$	369.1921	367.1776
–2C –4H	$C_{19}H_{22}N_4O_3$	355.1765	353.1619
+2H –2O	$C_{21}H_{28}N_4O$	353.2336	351.2190
–O	$C_{21}H_{26}N_4O_2$	367.2129	365.1983
+2H –O	$C_{21}H_{28}N_4O_2$	369.2285	367.2140
+O	$C_{21}H_{26}N_4O_4$	399.2027	397.1881
+6C +9H +6O	$C_{27}H_{35}N_4O_9$	559.2399	-
+10C +16H +2N +4O +S	$C_{31}H_{42}N_6O_7S$	643.2908	641.2763
+5C +8H +O +S	$C_{26}H_{34}N_4O_4S$	499.2374	497.2228
–3C –6H	$C_{18}H_{20}N_4O_3$	341.1608	339.1463
–4C –8H	$C_{17}H_{18}N_4O_3$	327.1452	325.1306
–4C –9H –N +O	$C_{17}H_{37}N_3O_4$	328.1292	326.1146
–4C –11H –N +O	$C_{17}H_{15}N_3O_4$	326.1135	324.0990
–4C –11H –N +2O	$C_{17}H_{15}N_3O_5$	342.1084	340.0939
+5C +6H +6O	$C_{26}H_{32}N_4O_9$	545.2242	543.2097
–C –2H +3O +S	$C_{20}H_{24}N_4O_6S$	449.1489	447.1344
+2C –H –N +7O	$C_{23}H_{25}N_3O_{10}$	504.1613	502.1467
–4C –9H –N +4O +S	$C_{17}H_{17}N_3O_7S$	408.0860	406.0714
+2C +4H –O	$C_{23}H_{30}N_4O_2$	395.2442	393.2296
+2H +2O	$C_{21}H_{28}N_4O_5$	417.2132	415.1987
–2H	$C_{21}H_{24}N_4O_3$	381.1921	379.1776
–2H +O	$C_{21}H_{24}N_4O_4$	397.1870	395.1725
–C –2H +O	$C_{20}H_{24}N_4O_4$	385.1870	383.1725
–5C –11H –N +O	$C_{16}H_{15}N_3O_4$	314.1135	312.0990
–5C –13H –N +O	$C_{16}H_{13}N_3O_4$	312.0979	310.0833
–2C –4H +O	$C_{19}H_{22}N_4O_4$	371.1714	369.1568
+4C +5H +6O	$C_{25}H_{31}N_4O_9$	531.2086	-
+2O	$C_{21}H_{26}N_4O_5$	415.1976	413.1830
+6C +8H +7O	$C_{27}H_{34}N_4O_{10}$	575.2348	573.2202
+4O +S	$C_{21}H_{26}N_4O_7S$	479.1595	477.1449
–4C –9H –N +2O	$C_{17}H_{17}N_3O_5$	344.1241	342.1095
+6C +9H +7O	$C_{27}H_{35}N_4O_{10}$	575.2348	-

Table S2-3. Inclusion list used during liquid chromatography-high-resolution tandem mass spectrometry (LC-HRMS/MS) for etodesnitazene metabolite identification

**Etodesnitazene**			
**Transformation**	**Elemental composition**	**$[M+H]^+$ *m/z***	**$[M-H]^-$ *m/z***
Parent (Etodesnitazene)	$C_{22}H_{29}N_3O$	352.2383	350.2238
–2C –4H	$C_{20}H_{25}N_3O$	324.2070	322.1925
+O	$C_{22}H_{29}N_3O_2$	368.2333	366.2187
–4C –8H	$C_{18}H_{21}N_3O$	296.1757	294.1612
–4C –9H –N +O	$C_{18}H_{20}N_2O_2$	297.1598	295.1452
–4C –11H –N +O	$C_{18}H_{18}N_2O_2$	295.1441	293.1296
–4C –11H –N +2O	$C_{18}H_{18}N_2O_3$	311.1390	309.1245
+4C +4H +6O	$C_{26}H_{33}N_3O_7$	500.2391	498.2246
–2C –4H +3O +S	$C_{20}H_{25}N_3O_4S$	404.1639	402.1493
+2C –H –N +7O	$C_{24}H_{28}N_2O_8$	473.1918	471.1773
–4C –9H –N +4O +S	$C_{18}H_{20}N_2O_5S$	377.1166	375.1020
+2H +2O	$C_{22}H_{31}N_3O_3$	386.2438	384.2293
–2H	$C_{22}H_{27}N_3O$	350.2227	348.2081
–2H +O	$C_{22}H_{27}N_3O_2$	366.2176	364.2031
–2C –4H +O	$C_{20}H_{25}N_3O_2$	340.2020	338.1874
–6C –13H –N +O	$C_{16}H_{16}N_2O_2$	269.1285	267.1139
–6C –15H –N +O	$C_{16}H_{14}N_2O_2$	267.1128	265.0983
+2O	$C_{22}H_{29}N_3O_3$	384.2282	382.2136
+6C +8H +7O	$C_{28}H_{37}N_3O_8$	544.2653	542.2508
+4O +S	$C_{22}H_{29}N_3O_5S$	448.1901	446.1755
–4C –9H –N +2O	$C_{18}H_{20}N_2O_3$	313.1547	311.1401
+10C +17H +3N +7O +S	$C_{32}H_{46}N_6O_8S$	675.3171	673.3025

Table S2-4. Inclusion list used during liquid chromatography-high-resolution tandem mass spectrometry (LC-HRMS/MS) for metodesnitazene metabolite identification

**Metodesnitazene**			
**Transformation**	**Elemental composition**	**$[M+H]^+$ *m/z***	**$[M-H]^-$ *m/z***
Parent (Metodesnitazene)	$C_{21}H_{27}N_3O$	338.2227	336.2081
–C –2H	$C_{20}H_{25}N_3O$	324.2070	322.1925
–2C –4H	$C_{19}H_{23}N_3O$	310.1914	308.1768
+O	$C_{21}H_{27}N_3O_2$	354.2176	352.2031
–3C –6H	$C_{18}H_{21}N_3O$	296.1757	294.1612
–4C –8H	$C_{17}H_{19}N_3O$	282.1601	280.1455
–4C –9H –N +O	$C_{17}H_{18}N_2O_2$	283.1441	281.1296
–4C –11H –N +O	$C_{17}H_{16}N_2O_2$	281.1285	279.1139
–4C –11H –N +2O	$C_{17}H_{16}N_2O_3$	297.1234	295.1088
+5C +6H +6O	$C_{26}H_{33}N_3O_7$	500.2391	498.2246
–C –2H +3O +S	$C_{20}H_{25}N_3O_4S$	404.1639	402.1493
+2C –H –N +7O	$C_{23}H_{26}N_2O_8$	459.1762	457.1616
–4C –9H –N +4O +S	$C_{17}H_{18}N_2O_5S$	363.1009	361.0864
+2H +2O	$C_{21}H_{29}N_3O_3$	372.2282	370.2136
–2H	$C_{21}H_{25}N_3O$	336.2070	334.1925
–2H +O	$C_{21}H_{25}N_3O_2$	352.2020	350.1874
–C –2H +O	$C_{20}H_{25}N_3O_2$	340.2020	338.1874
–5C –11H –N +O	$C_{16}H_{16}N_2O_2$	269.1285	267.1139
–5C –13H –N +O	$C_{16}H_{14}N_2O_2$	267.1128	265.0983
–2C –4H +O	$C_{19}H_{23}N_3O_2$	326.1863	324.1718
+2O	$C_{21}H_{27}N_3O_3$	370.2125	368.1980
+6C +8H +7O	$C_{27}H_{35}N_3O_8$	530.2497	528.2351
+4O +S	$C_{21}H_{27}N_3O_5S$	434.1744	432.1599
–4C –9H –N +2O	$C_{17}H_{18}N_2O_3$	299.1390	297.1245
+10C +17H +3N +7O +S	$C_{31}H_{44}N_6O_8S$	661.3014	659.2869
