# Supplementary material for: Human metabolism of four synthetic benzimidazole opioids: isotonitazene, metonitazene, etodesnitazene, and metodesnitazene
Source: Arch Toxicol. 2024 Apr 6;98(7):2101–16. doi: 10.1007/s00204-024-03735-0 (PMC11169013; doi:10.1007/s00204-024-03735-0)
Supplement: Supplementary file 3 — Supplementary file3 (PDF 201 KB) [file 204_2024_3735_MOESM3_ESM.pdf]

Table S3-1. Compound Discoverer processing settings for generating isotonitazene putative metabolites

**Isotonitazene**	
**Phase I reactions**	Deethylation (–2C –5H → +H)<br>Deisopropylation (–3C –7H → +H)<br>Desaturation (–2H → ø)<br>Dihydrodiol formation (ø → +2H +2O)<br>Nitro-reduction (–2O → +2H)<br>Oxidation (ø → +O)<br>Oxidative deamination to alcohol (–2H –N → +H +O)<br>Oxidative deamination to ketone (–3H –N → +O)<br>Reduction (ø → 2H)
**Phase II reactions**	Acetylation (–H → +2C +3H +O)<br>Cysteine conjugation (–H → +3C +6H +N +2O +S)<br>Cysteine-Glycine conjugation (–H → +5C +9H +2N +3O +S)<br>Glucuronide conjugation (–H → +6C +9H +6O)<br>Glutathione conjugation (–H → +10C +16H +3N +6O +S)<br>Methylation (–H → +C +3H)<br>Sulfation (–H → +H +3O +S)
**Max number of dealkylations**	3
**Max number of phase II reactions**	2
**Max number of all steps**	5
**Adducts**	$[M+H]^+$<br>$[M-H]^-$

Table S3-2. Compound Discoverer processing settings for generating metonitazene putative metabolites

**Metonitazene**	
**Phase I reactions**	Demethylation (–C –3H → +H)<br>Deethylation (–2C –5H → +H)<br>Desaturation (–2H → ø)<br>Dihydrodiol formation (ø → +2H +2O)<br>Nitro-reduction (–2O → +2H)<br>Oxidation (ø → +O)<br>Oxidative deamination to alcohol (–2H –N → +H +O)<br>Oxidative deamination to ketone (–3H –N → +O)<br>Reduction (ø → 2H)
**Phase II reactions**	Acetylation (–H → +2C +3H +O)<br>Cysteine conjugation (–H → +3C +6H +N +2O +S)<br>Cysteine-Glycine conjugation (–H → +5C +9H +2N +3O +S)<br>Glucuronide conjugation (–H → +6C +9H +6O)<br>Glutathione conjugation (–H → +10C +16H +3N +6O +S)<br>Methylation (–H → +C +3H)<br>Sulfation (–H → +H +3O +S)
**Max number of dealkylations**	3
**Max number of phase II reactions**	2
**Max number of all steps**	5
**Adducts**	$[M+H]^+$<br>$[M-H]^-$

Table S3-3. Compound Discoverer processing settings for generating etodesnitazene putative metabolites

**Etodesnitazene**	
**Phase I reactions**	Deethylation (–2C –5H → +H)<br>Desaturation (–2H → ø)<br>Dihydrodiol formation (ø → +2H +2O)<br>Oxidation (ø → +O)<br>Oxidative deamination to alcohol (–2H –N → +H +O)<br>Oxidative deamination to ketone (–3H –N → +O)<br>Reduction (ø → 2H)
**Phase II reactions**	Acetylation (–H → +2C +3H +O)<br>Cysteine conjugation (–H → +3C +6H +N +2O +S)<br>Cysteine-Glycine conjugation (–H → +5C +9H +2N +3O +S)<br>Glucuronide conjugation (–H → +6C +9H +6O)<br>Glutathione conjugation (–H → +10C +16H +3N +6O +S)<br>Methylation (–H → +C +3H)<br>Sulfation (–H → +H +3O +S)
**Max number of dealkylations**	3
**Max number of phase II reactions**	2
**Max number of all steps**	5
**Adducts**	$[M+H]^+$<br>$[M-H]^-$

Table S3-4. Compound Discoverer processing settings for generating metodesnitazene putative metabolites

**Metodesnitazene**	
**Phase I reactions**	Demethylation (–C –3H → +H)<br>Deethylation (–2C –5H → +H)<br>Desaturation (–2H → ø)<br>Dihydrodiol formation (ø → +2H +2O)<br>Oxidation (ø → +O)<br>Oxidative deamination to alcohol (–2H –N → +H +O)<br>Oxidative deamination to ketone (–3H –N → +O)<br>Reduction (ø → 2H)
**Phase II reactions**	Acetylation (–H → +2C +3H +O)<br>Cysteine conjugation (–H → +3C +6H +N +2O +S)<br>Cysteine-Glycine conjugation (–H → +5C +9H +2N +3O +S)<br>Glucuronide conjugation (–H → +6C +9H +6O)<br>Glutathione conjugation (–H → +10C +16H +3N +6O +S)<br>Methylation (–H → +C +3H)<br>Sulfation (–H → +H +3O +S)
**Max number of dealkylations**	3
**Max number of phase II reactions**	2
**Max number of all steps**	5
**Adducts**	$[M+H]^+$<br>$[M-H]^-$
